# Evaluation of large language model-generated medical information on idiopathic pulmonary fibrosis

**DOI:** 10.3389/frai.2025.1618378

**Published:** 2025-09-24

**Authors:** Iván Cherrez-Ojeda, Björn Christian Frye, Andreas Hoheisel, Arturo Cortes-Telles, Karla Robles-Velasco, Heidegger N. Mateos-Toledo, Ricardo G. Figueiredo, Christopher J. Ryerson, Gabriela Rodas-Valero, Juan Carlos Calderón

**Affiliations:** ^1^Universidad Espíritu Santo, Samborondon, Ecuador; ^2^Institute for Allergology, Charité – Universitätsmedizin Berlin, Corporate Member of Freie Universität Berlin and Humboldt-Universität zu Berlin, Berlin, Germany; ^3^Respiralab Research Group, Guayaquil, Ecuador; ^4^Clinic of Pneumology, Medical Center—University of Freiburg, Freiburg, Germany; ^5^Faculty of Medicine, University of Freiburg, Freiburg, Germany; ^6^Clinica de Enfermedades Respiratorias, Hospital Regional de Alta Especialidad de la Peninsula de Yucatan—IMSS Bienestar, Merida, Mexico; ^7^Clínica de Enfermedades Respiratorias, Hospital Regional de Alta Especialidad de la Península de Yucatán – IMSS Bienestar, Mérida, Yucatán, Mexico; ^8^Programa de Pós-Graduação em Saúde Coletiva, Universidade Estadual de Feira de Santana, Feira de Santana, Brazil; ^9^Department of Medicine and Centre for Heart Lung Innovation, University of British Columbia, Vancouver, BC, Canada

**Keywords:** idiopathic pulmonary fibrosis, artificial intelligence, natural language processing, machine learning, large language models, health information systems, quality of health care, clinical decision-making

## Abstract

**Background:**

Idiopathic Pulmonary Fibrosis (IPF) information from AI-powered large language models (LLMs) like ChatGPT-4 and Gemini 1.5 Pro is unexplored for quality, reliability, readability, and concordance with clinical guidelines.

**Research question:**

What is the quality, reliability, readability, and concordance to clinical guidelines of LLMs in medical and clinically IPF-related content?

**Study design and methods:**

ChatGPT-4 and Gemini 1.5 Pro responses to 23 ATS/ERS/JRS/ALAT IPF guidelines questions were compared. Six independent raters evaluated responses for quality (DISCERN), reliability (JAMA Benchmark Criteria), readability (Flesch–Kincaid), and guideline concordance (0–4). Descriptive analysis, Intraclass Correlation Coefficient, Wilcoxon signed-rank test, and effect sizes (r) were calculated. Statistical significance was set at *p* < 0.05.

**Results:**

According to JAMA Benchmark, ChatGPT-4 and Gemini 1.5 Pro provided partially reliable responses; however, readability evaluations showed that both models were difficult to understand. The Gemini 1.5 Pro provided significantly better treatment information (DISCERN score: 56 versus 43, *p* < 0.001). Gemini had considerably higher international IPF guidelines concordance than ChatGPT-4 (median 3.0 [3.0–3.5] vs. 3.0 [2.5–3.0], *p* = 0.0029).

**Interpretation:**

Both models gave useful medical insights, but their reliability is limited. Gemini 1.5 Pro gave greater quality information than ChatGPT-4 and was more compliant with worldwide IPF guidelines. Readability analyses found that AI-generated medical information was difficult to understand, stressing the need to refine it.

**What is already known on this topic:**

Recent advancements in AI, especially large language models (LLMs) powered by natural language processing (NLP), have revolutionized the way medical information is retrieved and utilized.

**What this study adds:**

This study highlights the potential and limitations of ChatGPT-4 and Gemini 1.5 Pro in generating medical information on IPF. They provided partially reliable information in their responses; however, Gemini 1.5 Pro demonstrated superior quality in treatment-related content and greater concordance with clinical guidelines. Nevertheless, neither model provided answers in full concordance with established clinical guidelines, and their readability remained a major challenge.

**How this study might affect research, practice or policy:**

These findings highlight the need for AI model refinement as LLMs evolve as healthcare reference tools to help doctors and patients make evidence-based decisions.

## 1 Introduction

The Idiopathic Pulmonary Fibrosis (an Update) and Progressive Pulmonary Fibrosis in Adults: An Official ATS/ERS/JRS/ALAT Clinical Practice Guideline defines Idiopathic Pulmonary Fibrosis (IPF) as a chronic and progressive lung disease marked by unexplained fibrosis and scarring of lung tissue, leading to declining pulmonary function and poor prognosis (Raghu et al., 2022; Renzoni et al., 2021). Patients typically experience worsening dyspnea, persistent dry cough, and reduced pulmonary function, as reflected in decreased forced vital capacity (FVC) and reduced diffusion capacity of the lungs for carbon monoxide (DLCO; Renzoni et al., 2021; Wuyts et al., 2020). Given the rapid progression of IPF, early diagnosis is essential and is primarily based on clinical history, high-resolution computed tomography (HRCT), and the exclusion of alternative conditions, often negating the need for invasive biopsy (Raghu et al., 2022). Given the complexity of IPF, high-quality, reliable, accessible, consistent, and easy-to-understand medical information is crucial for both healthcare providers and patients.

Recent advancements in artificial intelligence (AI), particularly large language models (LLMs) utilizing natural language processing (NLP), have transformed how medical information is accessed (Zhang et al., 2025). AI models such as ChatGPT-4 and Gemini 1.5 Pro can generate structured, human-like responses to medical inquiries and have demonstrated high proficiency in medical question-answering tasks. ChatGPT-4 has achieved near-perfect accuracy in standardized medical exams, while Google’s Med-PaLM 2, a specialized AI model, has shown high precision in medical reasoning (Wang et al., 2024). However, AI-generated content is susceptible to errors, biases, and inconsistencies due to the vast and unverified nature of the datasets these models are trained on (Zhai et al., 2024). Despite the growing number of tools for evaluating the quality of AI-generated information, including the QAMAI methodology (Vaira et al., 2024), the METRIC-framework (Schwabe et al., 2024), and similar, there remains insufficient amount of standardized data on the validation of AI-generated responses, which raises concerns regarding their quality and reliability in medical decision-making for IPF care (Shiferaw et al., 2024). In clinical decision-making applications, inaccurate, low-quality, inconsistent, or biased information might have serious clinical effects. Unaddressed bias in medical AI can lead to questionable clinical choices and worsen healthcare inequities (Cross et al., 2024). ChatGPT-4 (OpenAI) is designed for broad knowledge retrieval and specializes at providing coherent, user-friendly responses, making it ideal for medical education, patient communication, and general information support (Alhur, 2024; Ray, 2023), whereas Gemini 1.5 Pro (Google DeepMind) is built on a multimodal architecture that can process and reason across text, images, and code, allowing for more complex cognitive activities including clinical reasoning, literature analysis, and early diagnostic support (Alhur, 2024; Sonoda et al., 2024). While ChatGPT improves accessibility and clarity in healthcare conversations, Gemini’s deeper reasoning capabilities and multimodal integration have outstanding potential for assisting doctors with complex decision-making and tailored care (Mihalache et al., 2024; Popa et al., 2024; Shiferaw et al., 2024).

Despite their potential, the reliability of these models in pulmonary diseases, particularly regarding IPF, remains largely untested. Given the life-threatening nature of IPF (Chen et al., 2024), inaccurate or misleading medical information could have significant consequences, highlighting the importance of assessing AI-generated responses for quality, readability, and concordance with the actual guidelines of diagnosis and management of IPF (Xu and Shuttleworth, 2024).

This study aims to compare ChatGPT-4 and Gemini 1.5 Pro in their ability to generate reliable medical information about IPF. Key areas of evaluation include quality, reliability, and readability using validated evaluation tools. The study also aims to determine whether these AI models consistently align with established clinical guidelines from the American Thoracic Society (ATS) with the ultimate goal of evidencing potential strengths and limitations of AI-generated medical content and its integration into clinical practice.

## 2 Materials and methods

### 2.1 Study design

This is a single-stage study that compares the medical information produced by the LLMs ChatGPT-4 (OpenAI) and Gemini 1.5 Pro (Google DeepMind). To start the study, an exploration of Idiopathic Pulmonary Fibrosis (an Update) and Progressive Pulmonary Fibrosis in Adults: An Official ATS/ERS/JRS/ALAT Clinical Practice Guideline was first carried out (Raghu et al., 2022; Raghu et al., 2018).

### 2.2 Question selection and data collection

Based on the information from the recommendations about the diagnosis and treatment of IPF, the radiological and histopathological features of a Usual Interstitial Pneumonia (UIP), diagnostic approach, evidence-based recommendations for treatment and management approach of IPF, and future directions we identified in total 24 “strong” recommendations that were transformed to question form since under that format the conversation was carried out with ChatGPT-4 and Gemini 1.5 to obtain a response from those LLMs. The 24 initial questions are provided in the Supplementary Table S1. These questions were shared individually with the independent reviewers, who, following a joint consensus process via an online meeting, decided to rethink the question structure in order to comprehensively address the potential questions that both physicians and patients may have about an IPF diagnosis. A total of 23 questions were selected from the Idiopathic Pulmonary Fibrosis (an Update) and Progressive Pulmonary Fibrosis in Adults: An Official ATS/ERS/JRS/ALAT Clinical Practice Guideline (Raghu et al., 2022). Additionally, diagnostic questions that had not changed since the 2018 ATS/ERS/JRS/ALAT Clinical Practice Guideline (Raghu et al., 2018) were included. These questions were developed and refined based on feedback exchanges and comments from independent reviewers with the study investigators.

**Figure 1:**
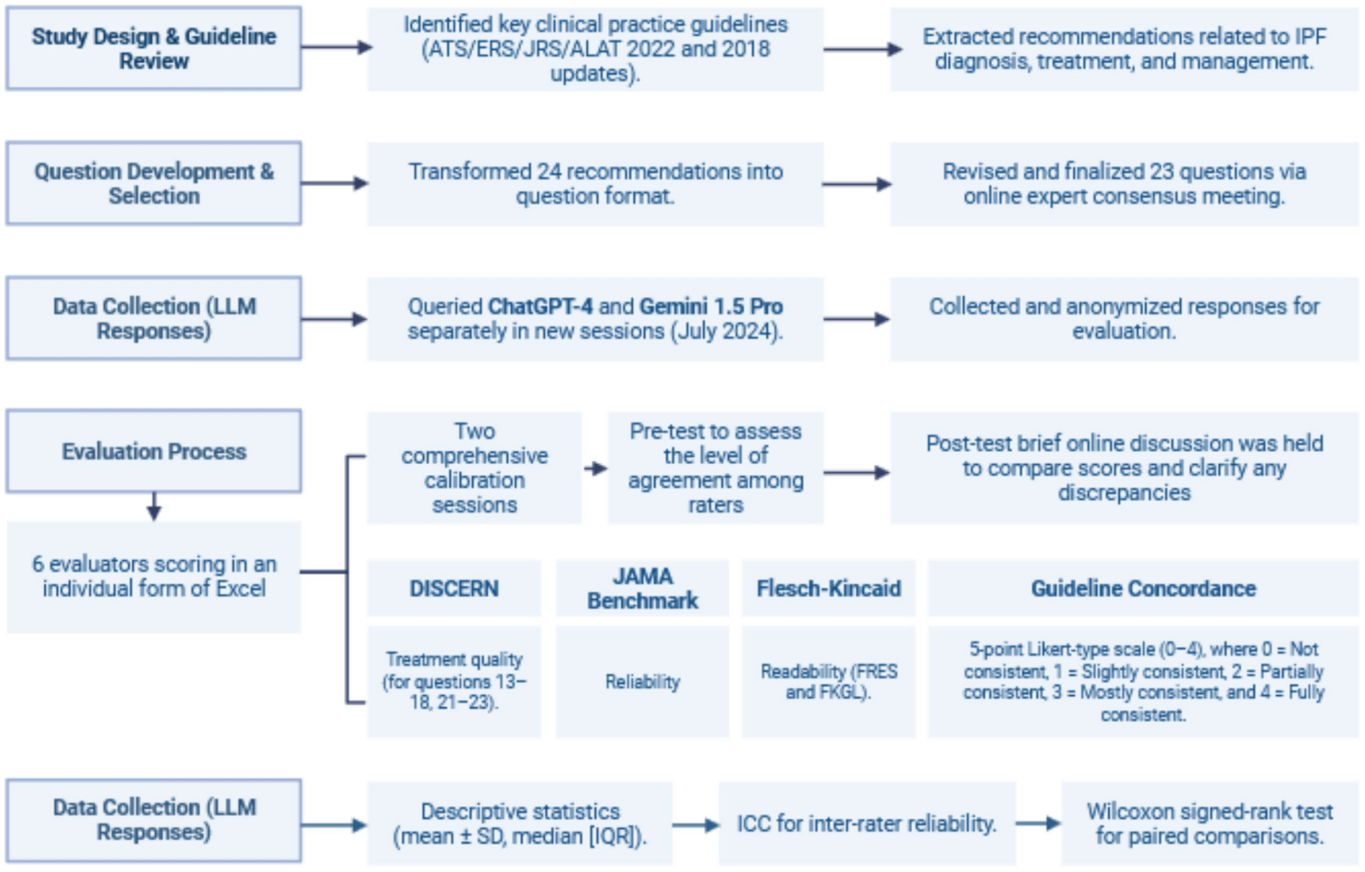
Overview of the methodological framework of this study.

In July 2024, each question was presented individually to both ChatGPT-4 and Gemini 1.5 Pro, with each query launching a new chat session to ensure that each response was created independently, avoiding potential contextual effects. The collected replies were structured, anonymized, and delivered to the selected panel of evaluators in the form of an Excel sheet, which was shared individually with each evaluator (see Figure 1).

### 2.3 Expert review and evaluation process

To enhance inter-rater consistency, all evaluators underwent a structured online training process led by an experienced instructor familiar with the assessment tools used in this study—namely, the DISCERN instrument, JAMA Benchmark criteria, Flesch–Kincaid Readability Tests, and domain-specific content analysis questions including concordance with clinical guidelines (Table 1).

**Table 1:** Validated tools used to evaluate large language models’ generated information.

Name	Components	Scoring
DISCERN (Charnock et al., 1999; Ozsoy, 2021)	Q1–Q8: Reliability Q9–Q15: Details of the information about treatment choices. Q16: Overall quality rating.	Maximum score: 80 points. Excellent quality: +63 Good quality: 51 to 62 Fair: 39 to 50 Poor: 27 to 38 Very poor: 16 to 26
JAMA Benchmark (Silberg et al., 1997)	Authorship Attribution Disclosure Currency	0 to 1.9 point: Insufficient information 2.0 to 3.9 points: Partially sufficient information 4 points: Completely sufficient information
Flesch–Kincaid Reading Ease Score (Flesch, 1948)	Flesch Reading Ease Score = 206.835–1.015 × (Total Words / Total Sentences) − 84.6 × (Total Syllables / Total Words)	Very Difficult: ≤29 Difficult: 30–49 Fairly Difficult: 50–59 Standard: 60–69 Fairly Easy: 70–79 Easy: 80–89 Very Easy: 90–100
Flesch–Kincaid Grade Level (Flesch, 1948)	Flesch–Kincaid Grade Level = 0.39 × (Total Words / Total Sentences) + 11.8 × (Total Syllables / Total Words) − 15.59	Grade 1–2: 1.0–2.9 Grade 3–4: 3.0–4.9 Grade 5–6: 5.0–6.9 Grade 7–8: 7.0–8.9 Grade 9–10.9: 9.0–10.9 Grade 11–12 (high school): 11.0–12.9 College (Undergraduate): 13.0–15.9 College Graduate / Professional: 16.0+

The training consisted of two comprehensive calibration sessions. During these sessions, evaluators received detailed instructions on how to apply each scoring tool consistently. Following the calibration sessions, a pre-test was administered to assess the level of agreement among raters. After completing the pre-test, a brief online discussion was held to compare scores and clarify any discrepancies, ensuring proper alignment and calibration among the reviewers.

Once calibration was confirmed, evaluators received the final Excel spreadsheet containing all the material to be assessed. The evaluations were conducted independently to minimize the risk of bias. The evaluators were blinded to the source model (ChatGPT-4 or Gemini 1.5 Pro) and each other’s scores. After a period of approximately 2 months, all ratings were collected. One investigator then compiled, cleaned, and coded the data to anonymize the results and prepare them for statistical analysis.

### 2.4 Assessment of information quality (DISCERN score)

The DISCERN (not an abbreviation) instrument, a validated tool designed to assess the quality of written consumer health information, was used to evaluate the accuracy and comprehensiveness of AI-generated treatment-related content (Charnock et al., 1999). DISCERN consists of 15 structured items divided into three sections, with an additional overall quality rating, yielding a maximum possible score of 80.

The first section evaluates the reliability of the information by determining whether clear objectives are stated, sources are cited, and content is presented objectively. The second section assesses the comprehensiveness of treatment-related details, including discussions on benefits, risks, and alternative management options. The final section consists of a single item that provides an overall assessment of the response’s quality. Given that DISCERN is primarily designed to evaluate treatment-related information, this tool was applied exclusively to responses addressing treatment recommendations (questions 13–18 and 21–23).

### 2.5 Assessment of reliability (JAMA benchmark criteria)

To assess content reliability, responses were analyzed using the JAMA (not an abbreviation) Benchmark Criteria, a widely used framework for evaluating the credibility of online health information (Rees et al., 2002). The JAMA Benchmark Criteria assess four fundamental domains: authorship, attribution, currency, and disclosure. Each response was assigned a score ranging from 0 to 4, with higher scores indicating greater concordance to quality benchmarks. Scores were categorized as insufficient information (0–1), partially sufficient information (2–3), or completely sufficient information (4) (Hoy et al., 2024).

### 2.6 Assessment of readability (Flesch–Kincaid readability tests)

Readability was assessed using two established metrics: the Flesch–Kincaid Reading Ease Score (FRES) and the Flesch–Kincaid Grade Level (FKGL; Flesch, 1948). The FRES assigns a numerical value ranging from 0 to 100, with higher scores indicating easier readability. The FKGL estimates the educational grade level required to understand the text. Both scores were calculated using an online Flesch–Kincaid calculator to maintain objectivity.^1^

### 2.7 Assessment of concordance with guidelines

A comparative content analysis of ChatGPT-4 and Gemini 1.5 assessed four primary domains: Definition, Diagnosis, Follow-up, and Treatment. Responses were evaluated by six raters using a 5-point Likert-type scale ranging from 0 to 4 to assess concordance with guideline recommendations, and median scores along with their interquartile ranges (IQR) were computed for each domain. The scale was defined as follows: 0 = Not consistent, the response contradicts or disregards established guideline recommendations, it provides misleading, irrelevant, or incorrect information with no alignment to evidence-based practices; 1 = Slightly consistent, the response shows minimal alignment with the guidelines, mentioning a related concept but missing critical aspects or including substantial inaccuracies; 2 = Partially consistent, the response incorporates some elements of the guidelines, but the information is incomplete, lacks detail, or includes notable errors or omissions that reduce its reliability. 3 = Mostly consistent, the response aligns well with the guidelines, covering most of the key recommendations accurately. Minor omissions, simplifications, or imprecisions may be present but do not significantly alter the overall correctness. 4 = Fully consistent, an answer that includes critical components to address the issue and tackles the most pertinent aspects in a concentrated and systematic manner, elucidating the information clearly, precisely, and methodically in relation to the guideline. Supplementary Table S2 shows the scores for each response provided by ChatGPT-4 and Gemini 1.5 Pro.

### 2.8 Statistical analysis

All statistical analyses were performed using Stata 18.0. Descriptive statistics were calculated according to the distribution of each variable: for parametric distributions, such as Flesch–Kincaid Grade Level (FKGL) scores, results are presented as mean ± standard deviation; for non-parametric distributions, including DISCERN and JAMA Benchmark scores, results are presented as median and interquartile range (IQR). To assess inter-rater reliability, the Intraclass Correlation Coefficient (ICC) was calculated based on six independent raters using a two-way random-effects model.

Concordance with guidelines was assessed using a 5-point Likert-type scale (0–4), where 0 = Not consistent, 1 = Slightly consistent, 2 = Partially consistent, 3 = Mostly consistent, and 4 = Fully consistent. As the data consisted of paired ordinal scores (0–4 scale), nonparametric testing was employed. Descriptive statistics were reported as median with IQR. Differences between paired scores were assessed using the Wilcoxon signed-rank test. Effect size (r) was calculated as *Z*/√*N* to quantify the magnitude of the observed differences, with thresholds of 0.1, 0.3, and 0.5 interpreted as small, medium, and large effects, respectively. Statistical significance was defined as a two-tailed *p* < 0.05. Additionally, boxplots were used for visual comparison, and Bland–Altman plots were generated to examine the bias between models.

### 2.9 Ethical considerations

This study did not include human participants, patient data, or direct medical interventions. Therefore, formal ethical approval was not required. However, principles of responsible AI research were upheld throughout the study, including the anonymization of AI-generated responses before evaluation and ensuring that all assessments were conducted independently by expert reviewers. Transparency in reporting results and concordance with objective evaluation standards were also prioritized to maintain scientific integrity.

## 3 Results

### 3.1 Overall performance of AI-generated responses

The 23 queries were classified into four categories: definition, diagnosis, treatment, and follow-up (Tables 2, 3). Across all categories, the JAMA Benchmark evaluations demonstrated that both ChatGPT-4 and Gemini 1.5 Pro provided partially sufficient information, with a median score of 2 in both models, however, there were no statistically significant differences between the two AI systems (*p* = 0.24). Readability assessments showed that responses generated by both models were classified as very difficult to read, requiring at least a college graduate-level education for full comprehension, as indicated by the FKGL. In terms of concordance with guidelines, we identified both models were mostly consistent (score of 3) with IPF guidelines. We are presenting the scores per each category assessed in the study below.

**Table 2:** Scores obtained for information reliability and readability.

Category	Question	ChatGPT-4 JAMA Benchmark^b^			Gemini 1.5 Pro JAMA Benchmark^b^			ChatGPT-4 Flesh Readability Assessment^c^			Gemini 1.5 Pro Flesh Readability Assessment^c^		
		Median	IQR	Interpretation	Median	IQR	Interpretation	Reading Ease Score^d^	Readability Level	Grade Level	Reading Ease Score	Readability Level	Grade Level
Definition	What is “clinically suspected idiopathic pulmonary fibrosis”?	2.5	3.0	Partially sufficient information	2	3	Partially sufficient information	16.9	Very difficult	College graduate	12.7	Very difficult	College graduate
	What does “likely idiopathic pulmonary fibrosis” mean?	0	2.0	Insufficient information	0.5	3	Insufficient information	47.3	Difficult	College	37	Difficult	College
Category overall, mean (SD) or median (IQR)		1.25	2.5	Insufficient information	1.25	1.5	Insufficient information	32.1 (21.5)	Difficult	College	24.9 (17.2)	Very difficult	College graduate
Diagnosis	What are the criteria for indeterminate idiopathic pulmonary fibrosis?	2.5	3	Partially sufficient information	2.5	3	Partially sufficient information	16.8	Very difficult	College graduate	6.8	Very difficult	College graduate
	Should patients with newly detected interstitial lung disease of unknown cause who are clinically suspected of having idiopathic pulmonary fibrosis undergo serological testing to exclude connective tissue diseases as a potential cause of their interstitial lung disease?	2.5	3	Partially sufficient information	0	3	Insufficient information	0.8	Very difficult	College graduate	1.6	Very difficult	College graduate
	What autoimmune serologies should be performed in a patient with suspected IPF?	2.5	3	Partially sufficient information	1.5	3	Insufficient information	11.6	Very difficult	College graduate	0	Very difficult	College graduate
	Should patients with newly detected interstitial lung disease of unknown cause who are clinically suspected of having idiopathic pulmonary fibrosis be the subject of multidisciplinary discussion for decision-making?	2.5	3	Partially sufficient information	1.5	3	Insufficient information	0	Very difficult	College graduate	0	Very difficult	College graduate
	Can multidisciplinary discussion be used in some clinical settings to make a diagnosis of idiopathic pulmonary fibrosis in patients with a radiological pattern of probable usual interstitial pneumonia without confirmation by lung biopsy?	2.5	3	Partially sufficient information	0	3	Insufficient information	11.4	Very difficult	College graduate	17.1	Very difficult	College graduate
	Should patients with newly detected interstitial lung disease of unknown cause who are clinically suspected of having idiopathic pulmonary fibrosis undergo cellular analysis of their bronchoalveolar fluid?	2.5	3	Partially sufficient information	1.5	3	Insufficient information	9.6	Very difficult	College graduate	0	Very difficult	College graduate
	For patients with newly detected interstitial lung disease of unknown cause who are clinically suspected of having idiopathic pulmonary fibrosis, should surgical lung biopsy be performed to ascertain the histopathology pattern of usual interstitial pneumonia?	2.5	3	Partially sufficient information	2	3	Partially sufficient information	18.4	Very difficult	College graduate	0	Very difficult	College graduate
	For patients with newly detected interstitial lung disease of unknown cause who are clinically suspected of having idiopathic pulmonary fibrosis, is transbronchial lung cryobiopsy a reasonable alternative to surgical lung biopsy to ascertain the histopathology pattern of usual interstitial pneumonia?	2.5	3	Partially sufficient information	2.5	3	Partially sufficient information	10.9	Very difficult	College graduate	9.9	Very difficult	College graduate
	Should patients who are clinically suspected of having idiopathic pulmonary fibrosis and have nondiagnostic findings in a transbronchial lung cryobiopsy undergo surgical lung biopsy?	2.5	3	Partially sufficient information	0.5	3	Insufficient information	19.2	Very difficult	College graduate	0	Very difficult	College graduate
	Should genomic classifier testing be performed for the purpose of identifying usual interstitial pneumonia in patients with interstitial lung disease of undetermined type who are undergoing any form of lung biopsy?	2.5	3	Partially sufficient information	2.5	3	Partially sufficient information	16.9	Very difficult	College graduate	0	Very difficult	College graduate
Category overall, mean (SD) or median (IQR)		2.5	-^a^	Partially sufficient information	1.5	2.1	Insufficient information	11.6 (6.8)	Very difficult	College graduate	3.5 (5.9)	Very difficult	College graduate
Treatment	Should patients with idiopathic pulmonary fibrosis and mild to moderate impairment in pulmonary function be treated with pirfenidone as a first line treatment?	2.5	3	Partially sufficient information	0	3	Insufficient information	23.5	Very difficult	College graduate	26	Very difficult	College graduate
	Should patients with idiopathic pulmonary fibrosis and mild to moderate impairment in pulmonary function be treated with nintedanib as a first line treatment?	2.5	3	Partially sufficient information	2.5	3	Partially sufficient information	21.5	Very difficult	College graduate	27.1	Very difficult	College graduate
	When should patients with idiopathic pulmonary fibrosis be treated with supplemental O2?	2.5	3	Partially sufficient information	2.5	3	Partially sufficient information	19.9	Very difficult	College graduate	27.2	Very difficult	College graduate
	Should patients with idiopathic pulmonary fibrosis and confirmed gastroesophageal reflux, with or without symptoms of gastroesophageal reflux disease, be treated with antacid medications to improve respiratory outcomes?	2.5	3	Partially sufficient information	2	3	Partially sufficient information	12.4	Very difficult	College graduate	23	Very difficult	College graduate
	Should patients with idiopathic pulmonary fibrosis and confirmed gastroesophageal reflux, with or without symptoms of gastroesophageal reflux disease, be referred for antireflux surgery to improve respiratory outcomes?	0	3	Insufficient information	2.5	3	Partially sufficient information	0	Very difficult	College graduate	25.7	Very difficult	College graduate
	Which patients with idiopathic pulmonary fibrosis should be referred for pulmonary rehabilitation?	2.5	3	Partially sufficient information	2.5	3	Partially sufficient information	0.8	Very difficult	College graduate	6.4	Very difficult	College graduate
	Which patients with idiopathic pulmonary fibrosis should be referred for lung transplantation?	2.5	3	Partially sufficient information	2.5	3	Partially sufficient information	15.3	Very difficult	College graduate	11.3	Very difficult	College graduate
	Should patients with acute exacerbation of idiopathic pulmonary fibrosis be treated with corticosteroids?	2.5	3	Partially sufficient information	2.5	3	Partially sufficient information	8.4	Very difficult	College graduate	15.7	Very difficult	College graduate
	Should patients with idiopathic pulmonary fibrosis and respiratory failure be treated with mechanical ventilation?	2.5	3	Partially sufficient information	2	3	Partially sufficient information	30.8	Difficult	College	21.6	Very difficult	College graduate
Category overall, mean (SD) or median (IQR)		2.5	-^a^	Partially sufficient information	2.5	0.5	Partially sufficient information	14.7 (10.4)	Very difficult	College graduate	20.4 (7.6)	Very difficult	College graduate
Follow up	How frequently should patients with idiopathic pulmonary fibrosis undergo high-resolution computed tomography of the chest for monitoring purposes?	2.5	3	Partially sufficient information	2.5	3	Partially sufficient information	27.4	Very difficult	College graduate	0	Very difficult	College graduate
	How frequently should patients with idiopathic pulmonary fibrosis undergo pulmonary function testing with spirometry and DLCO?	2.5	3	Partially sufficient information	2.5	3	Partially sufficient information	23.5	Very difficult	College graduate	0	Very difficult	College graduate
Category overall, mean (SD) or median (IQR)		2.5	-^a^	Partially sufficient information	2.5	-^a^	Partially suficiente information	25.5 (2.8)	Very difficult	College graduate	0	Very difficult	College graduate
Total mean (SD) or median (IQR)		2.5	-^a^	Partially sufficient information	2	1	Partially sufficient information	15.8 (10.9)	Very difficult	College graduate	11.7 (11.8)	Very difficult	College graduate

a The distribution is perfectly non-variable, so the IQR is 0.

b The data follows a non-parametric distribution, values expressed in median (IQR).

c The data follows parametric distribution, values expressed in mean (SD).

d The values presented correspond to the single score obtained by the Flesch–Kincaid readability formula.

**Table 3:** Scores obtained for information quality according to DISCERN.

Question	DISCERN					
	ChatGPT-4			Gemini 1.5 Pro		
	Median	IQR	Interpretation	Median	IQR	Interpretation
Should patients with idiopathic pulmonary fibrosis and mild to moderate impairment in pulmonary function be treated with pirfenidone as a first line treatment?	49.5	18	Fair quality	57.5	4	Good quality
Should patients with idiopathic pulmonary fibrosis and mild to moderate impairment in pulmonary function be treated with nintedanib as a first line treatment?	47	22	Fair quality	59	7	Good quality
When should patients with idiopathic pulmonary fibrosis be treated with supplemental O2?	41.5	18	Fair quality	43.5	6	Fair quality
Should patients with idiopathic pulmonary fibrosis and confirmed gastroesophageal reflux, with or without symptoms of gastroesophageal reflux disease, be treated with antacid medications to improve respiratory outcomes?	41	24	Fair quality	57	15	Good quality
Should patients with idiopathic pulmonary fibrosis and confirmed gastroesophageal reflux, with or without symptoms of gastroesophageal reflux disease, be referred for antireflux surgery to improve respiratory outcomes?	39	22	Fair quality	49	10	Fair quality
Which patients with idiopathic pulmonary fibrosis should be referred for pulmonary rehabilitation?	41	19	Fair quality	57	5	Good quality
Which patients with idiopathic pulmonary fibrosis should be referred for lung transplantation?	38.5	13	Poor quality	44	13	Fair quality
Should patients with acute exacerbation of idiopathic pulmonary fibrosis be treated with corticosteroids?	43	18	Fair quality	63.5	5	Excellent quality
Should patients with idiopathic pulmonary fibrosis and respiratory failure be treated with mechanical ventilation?	42	19	Fair quality	56	7	Good quality
Overall	43	22	Fair quality	56	12	Good quality

### 3.2 Definition

**Figure 2:**
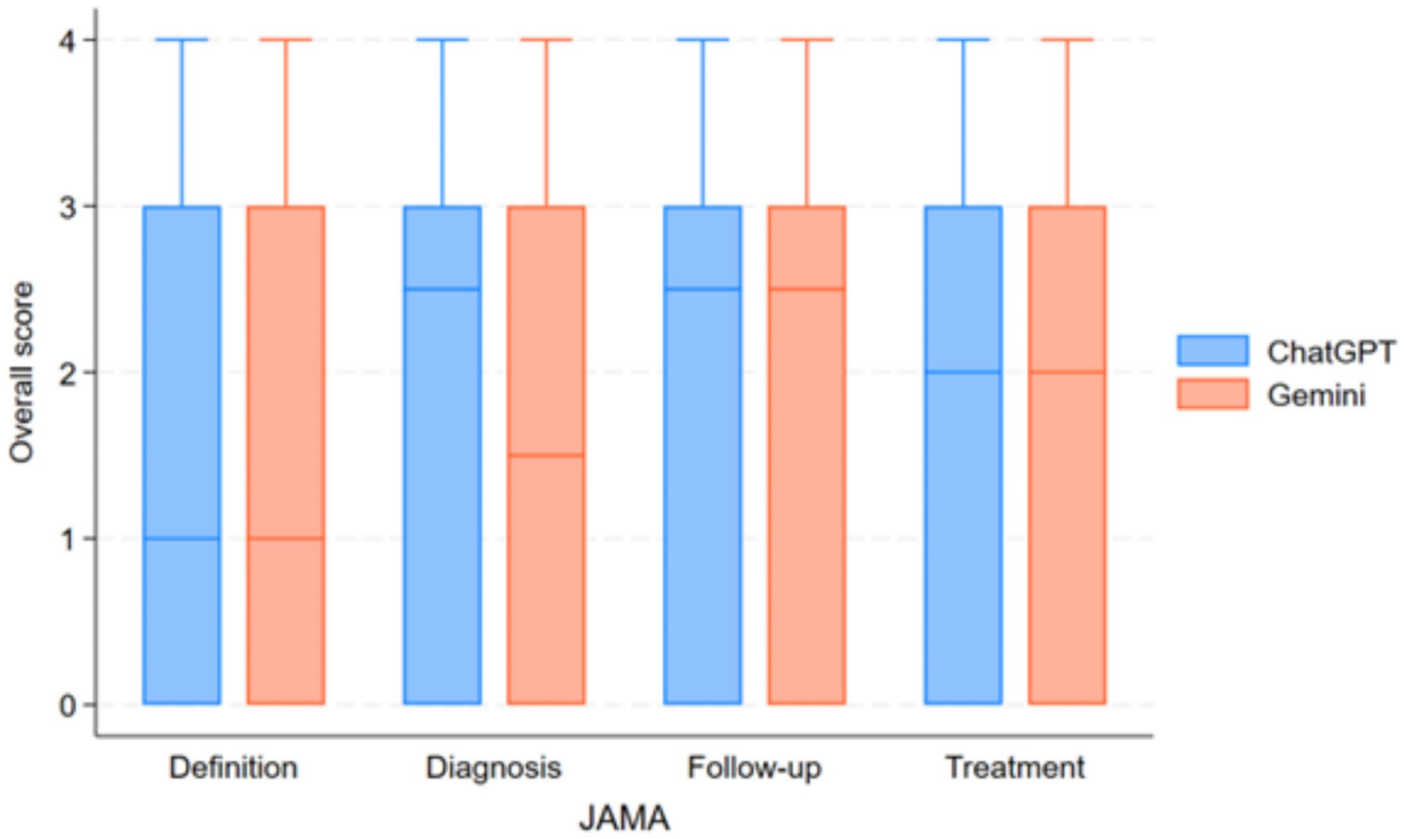
Comparison of reliability scores (JAMA benchmark criteria) between ChatGPT-4 and Gemini 1.5 Pro across different IPF-related question categories.

For questions assessing the definition of IPF, ChatGPT-4 and Gemini 1.5 Pro both received a median JAMA Benchmark score of 1.25, indicating that the information had insufficient attributes of reliability, without a statistically significant difference between the two models (*p* = 0.78; Figure 2).

**Figure 3:**
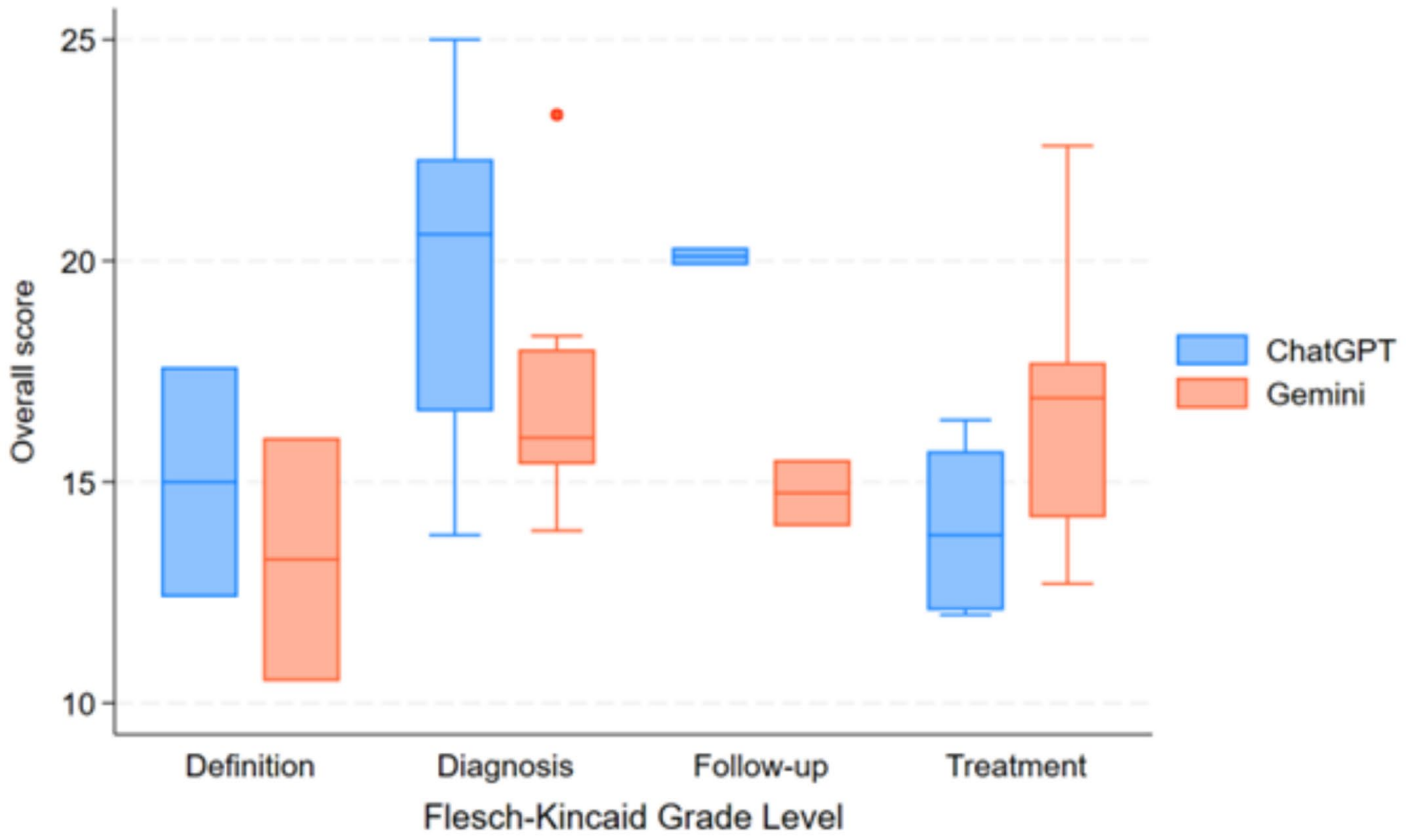
Comparison of readability scores (Flesch–Kincaid grade level) between ChatGPT-4 and Gemini 1.5 pro across different IPF-related question categories.

Readability assessment using FRES indicated that ChatGPT-4 responses were slightly easier to read (mean FRES score: 32.1 ± 21.5, “Difficult”) compared to Gemini 1.5 Pro (mean FRES score: 24.9 ± 17.2, “Very Difficult”), and both required at least a college reading level, as reflected by FKGL scores (Figure 3).

### 3.3 Diagnosis

In the diagnosis category, ChatGPT-4 achieved a median JAMA Benchmark score of 2.5, which was classified as partially sufficient information, meanwhile, Gemini 1.5 Pro received a lower median score of 1.5, falling into the insufficient information category; nevertheless, this difference was not statistically significant (*p* = 0.078). Readability analysis indicated that responses from both models were very difficult to read, with college graduate-level comprehension required to fully understand the content.

### 3.4 Treatment

The quality of treatment-related responses was evaluated using both the DISCERN scale and JAMA Benchmark Criteria. ChatGPT-4 received a mean DISCERN score of 43 (reflecting fair quality), whereas Gemini 1.5 Pro achieved a significantly higher mean score of 56 (considered good quality). The difference was statistically significant (*p* < 0.001), indicating that Gemini 1.5 Pro generated higher-quality treatment information compared to ChatGPT-4.

Despite the difference in information quality, both models received a median JAMA Benchmark score of 2.5, classifying their content as partially sufficient information with no significant difference (*p* = 0.89). Readability remained a challenge for both models, with FRES scores indicating that responses were very difficult to read and required college graduate-level comprehension, as per FKGL scores.

### 3.5 Follow-up

For questions regarding patient follow-up, both ChatGPT-4 and Gemini 1.5 Pro received a median JAMA Benchmark score of 2.5, denoting partially sufficient information, without a significant difference between groups (*p* = 1.0). Readability analysis again showed that both AI-generated responses were very difficult to read, requiring college graduate-level comprehension.

### 3.6 Assessment of concordance with guidelines

**Figure 4:**
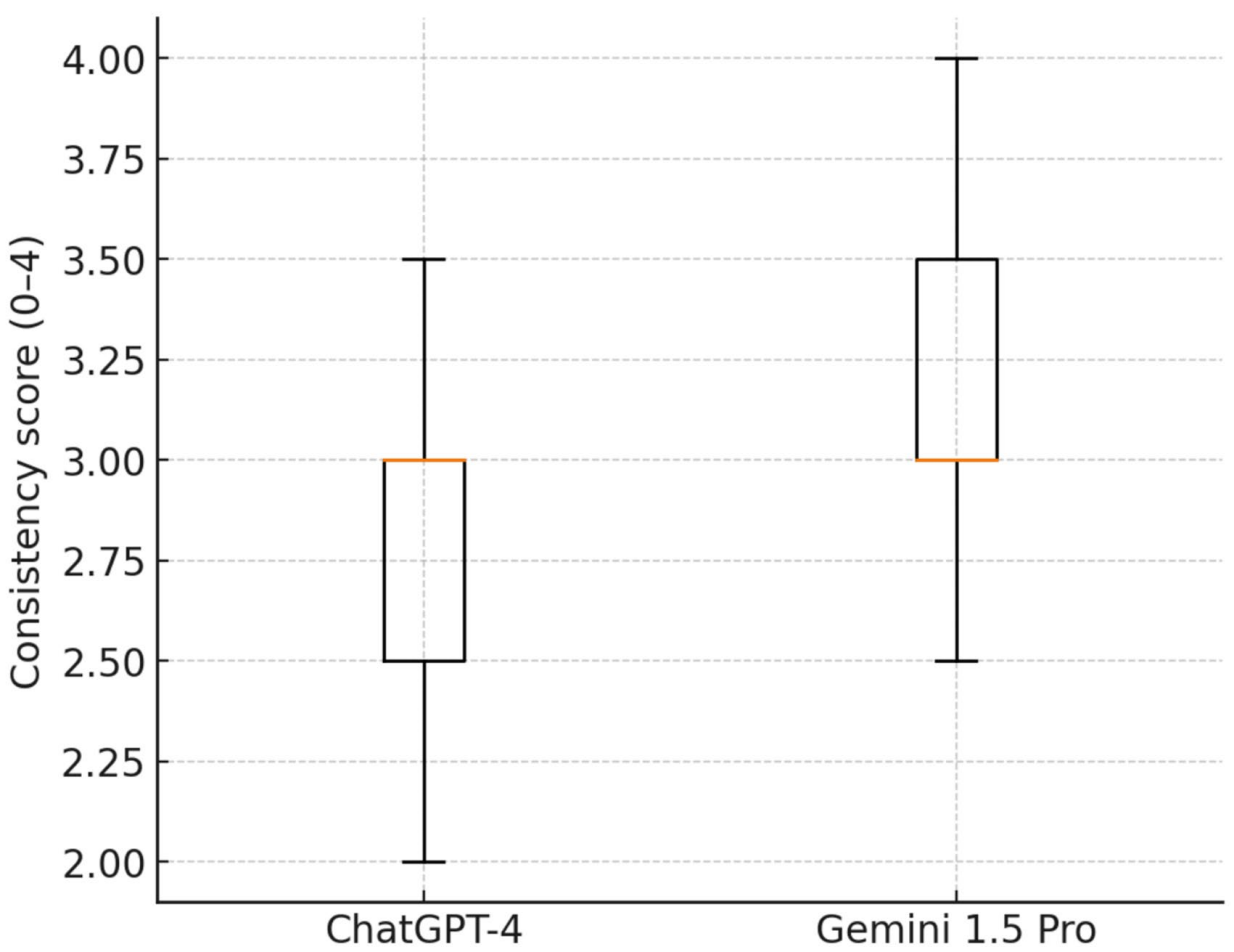
Boxplot showing a comparison of guideline concordance scores between ChatGPT-4 and Gemini 1.5 Pro.

**Figure 5:**
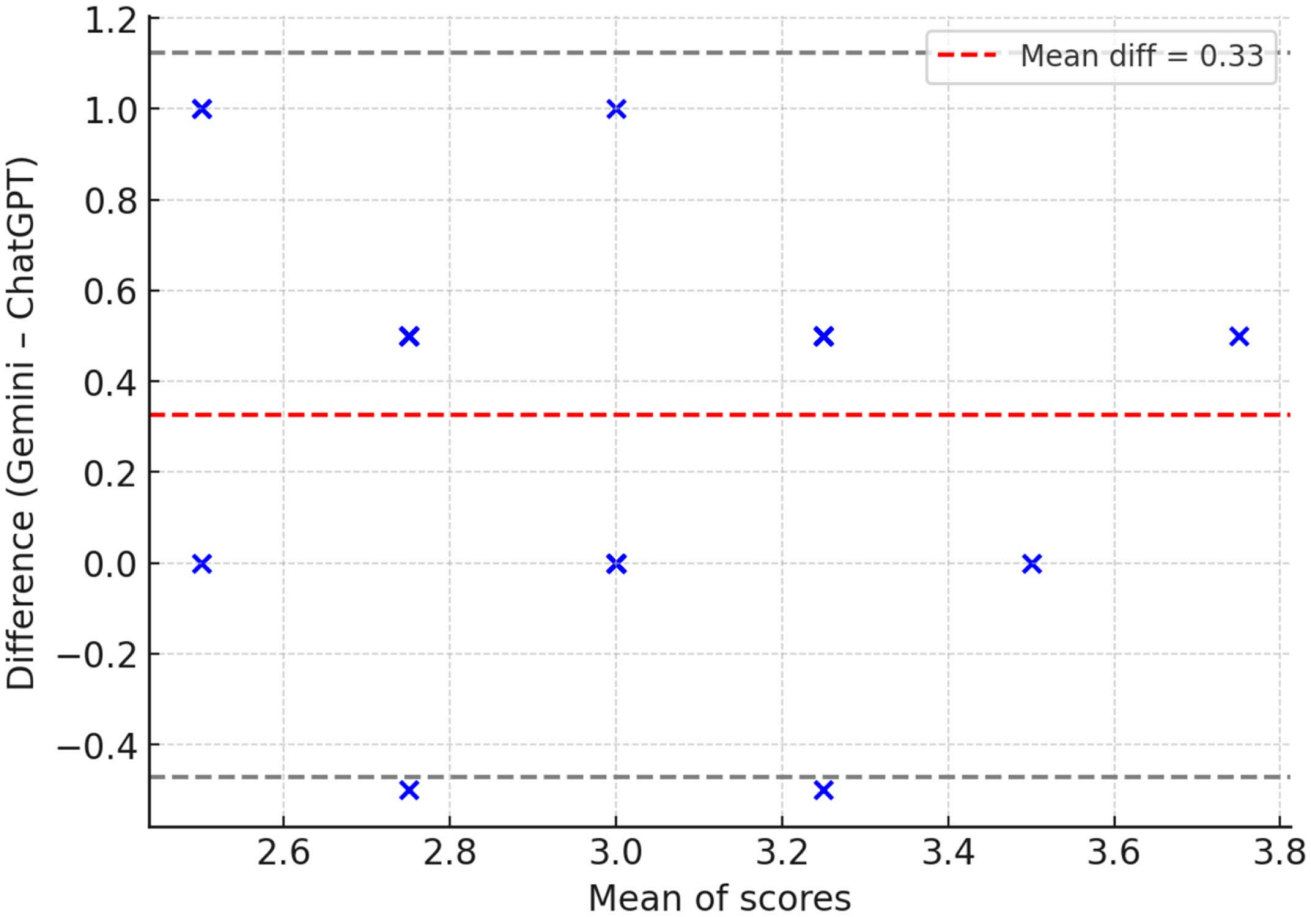
Bland–Altman plot: agreement between models across 23 paired items. The mean difference (red dashed line) was +0.33 in favor of Gemini, indicating a systematic bias toward higher scores. Most differences fell within the 95% limits of agreement (gray dashed lines), confirming that this bias was consistent across items.

When comparing guideline concordance across 23 clinical questions on IPF, both ChatGPT-4 and Gemini 1.5 Pro showed generally acceptable alignment, with substantial variances in several domains. Overall, Gemini 1.5 Pro received slightly higher scores, particularly for diagnosis and treatment recommendations, where its responses were more commonly classified as “mostly consistent” or “fully consistent” with established guidelines (Figure 4). For example, Gemini surpassed ChatGPT-4 in addressing the function of complex diagnostic pathways such as serological testing, bronchoalveolar lavage, surgical lung biopsy, cryobiopsy, and genetic classifier testing. In terms of treatment, Gemini provided more consistent results with pirfenidone, nintedanib, oxygen supplementation, and gastroesophageal reflux disease care, whereas ChatGPT-4 produced only partially consistent results. Questions about multidisciplinary discussion, referral for lung transplantation, acute exacerbation therapy with corticosteroids, and the use of mechanical ventilation showed areas of agreement between models, with both systems generally congruent with current guidelines. Taken together, Gemini 1.5 Pro achieved significantly higher guideline consistency scores compared with ChatGPT-4 (median [IQR]: 3.0 [3.0–3.5] vs. 3.0 [2.5–3.0]; Wilcoxon signed-rank test: W = 14.0, *p* = 0.0029; effect size r = −0.79). The mean difference was +0.33 in favor of Gemini, indicating a large and clinically relevant effect, see Table 4 and Figure 5.

**Table 4:** Comparison of concordance with guidelines scores between ChatGPT-4 and Gemini 1.5 Pro.

Variable	ChatGPT-4 (n = 23)	Gemini 1.5 Pro (n = 23)	Test statistic	*p*-value	Effect size (r)
Median (IQR)	3.0 (0.5)	3.0 (0.5)			
Range	2.0–3.5	2.5–4.0			
Wilcoxon signed-rank test (W)			14.0	0.0029	−0.79 (large)
Mean difference (Gemini—ChatGPT)					+0.33

Scores ranged from 0 (not consistent) to 4 (fully consistent). Data are shown as median with interquartile range (IQR), and range. Differences between models were evaluated using the Wilcoxon signed-rank test.

Distribution of concordance with guidelines scores (0–4 scale) for both models. Gemini 1.5 Pro demonstrated higher overall scores, with maximum values up to 4.0 compared with 3.5 for ChatGPT-4. While medians were equal (3.0), Gemini displayed a right-shifted distribution, reflecting more frequent higher scores.

### 3.7 Intraclass correlation and reviewer agreement

The intraclass correlation coefficient (ICC) for content analysis scores was of 0.507 for ChatGPT-4 and 0.544 for Gemini 1.5 Pro, reflecting a moderate level of agreement.

## 4 Discussion

The evaluation of large language models (LLMs) in healthcare has grown more extensive in recent years, alongside an increasing interest in utilizing these tools inside clinical environments (Thirunavukarasu et al., 2023). The specialties where evaluations of performance of LLM has been carried out, involve generic health care, internal medicine, surgery, and ophthalmology, however in the pulmonology field, specifically in IPF there are still gaps of knowledge(Bedi et al., 2025). In high-stakes condition such as idiopathic pulmonary fibrosis (IPF), the consequences of AI models offering inaccurate or incomplete suggestions could be severe. Bedi et al. recommend evaluations must incorporate authentic patient information, measure bias, encompass a broader spectrum of medical roles and specialties, and present standardized performance metrics (Bedi et al., 2025). In our study, we performed a comparative evaluation using 3 different metrics for assessing reliability (JAMA benchmark; Hoy et al., 2024), readability (Flesch Kincaid; Flesch, 1948), and quality (DISCERN; Rees et al., 2002) of online information. Overall, our analysis revealed that while both ChatGPT-4 and Gemini 1.5 Pro provided partially sufficient and difficult-to-read responses across domains, notable differences emerged in treatment-related content, where Gemini 1.5 Pro achieved significantly higher quality scores on the DISCERN scale. In contrast, no significant differences were observed between models in the domains of definition, diagnosis, or follow-up, as reflected by similar JAMA Benchmark scores.

The results indicate that both ChatGPT-4 and Gemini 1.5 Pro produced partially sufficient information across all categories evaluated. While both models showed an overall alignment with established medical concepts, the presence of insufficient and partially sufficient responses suggests limitations in their ability to consistently provide high-quality medical information.

A related study revealed similar results regarding the reliability of ChatGPT replies in urticaria, indicating a lack of reliable responses about the assessment and monitoring of this condition (Cherrez-Ojeda et al., 2025). The accuracy of responses produced by LLMs depends on the quantity, quality, and characteristics of the training data employed. If the original data lacks this information, the LLM’s answer will also be devoid of it.(Eggmann et al., 2023). Walker et al.´s (Walker et al., 2023) demonstrated that the majority of responses from LLMs were devoid of information regarding the sources, including the issuing bodies, individuals, or institutions responsible for the information’s generation, which subsequently diminished the reliability scores evaluated by the JAMA benchmark tool.

Our investigation indicated that ChatGPT-4 provided replies of fair quality, but Gemini 1.5 Pro demonstrated superior performance with good outcomes. Zhou et al.(Zhou et al., 2025) reported analogous results when evaluating DeepSeek and ChatGPT, with all models exhibiting DISCERN scores beneath 60, signifying merely “fair” information quality, primarily due to insufficient source citations.

The findings indicate that there remains potential for enhancement in the LLMs, particularly concerning the lack of detailed information regarding the sources of the data provided and the dates of the responses generated. This deficiency may undermine user trust and highlights a comparative weakness that the developers of these LLMs have yet to address (Dastani et al., 2025; Reyhan et al., 2024).

Readability consistently posed a limitation for both models, with responses requiring college-level comprehension. The readability of AI-generated medical content is a critical factor to widespread use for both healthcare professionals, healthcare students, and patients. This importance is highlighted by studies where students have shown interest in learning about the applications of ChatGPT in particular cases of medical practice, followed by homework support and understanding the benefits and limits (Cherrez-Ojeda et al., 2024).

Studies have evidenced that AI-generated content often requires a high level of reading proficiency, which can limit its accessibility. For instance, a study by Golan et al. (2023)evaluated ChatGPT’s proficiency in utilizing the DISCERN tool and found that the generated content was complex and not easily understandable for the general public. Similarly, Malik et al. (2023) explored students’ perceptions of AI usage in academic essay writing and highlighted challenges in readability and comprehension. These findings suggest that while AI-generated content may be valuable for clinicians and researchers, it is not well-optimized for broader public consumption (Cherrez-Ojeda et al., 2024). The complexity of the text may act as a barrier to patient education, particularly for individuals with lower health literacy.

Despite these challenges, some studies have explored methods to improve the readability of AI-generated medical information. For example, a study published by Akkan and Seyyar (2025) investigated the role of prompt wording on ChatGPT’s responses and found that using conversational prompts can enhance readability. The authors concluded that clinicians and content creators should consider this approach when using AI for patient education to optimize comprehension.

In order to achieve more suitable models for healthcare applications, researchers and developers continue to refine LLM systems through specialized tuning techniques. However, deploying these generic models for patient information remains challenging because their training data may not contain vetted medical information. To address this limitation, fine-tuning generic LLMs with domain-specific information represents a viable solution. Biomed-BERT and BioGPT, for example, were trained using peer-reviewed literature, while Med-PaLM was trained using clinical question databases (Singhal et al., 2023). Biomedical natural language processing (NLP) tasks have been significantly improved by these approaches (Sevgi et al., 2024).

Another key aspect when evaluating AI-generated medical content is that it should have concordance with clinical guidelines (Salybekov et al., 2024). Gemini 1.5 Pro showed superior concordance with IPF guidelines compared to ChatGPT-4, especially in diagnosis and treatment domains, where it more accurately addressed complex diagnostic tools and therapeutic options. While both models performed similarly in areas such as multidisciplinary care and acute exacerbation management, the overall effect size indicated a clear advantage for Gemini, highlighting its greater clinical reliability.

A systematic review by Kolbinger et al. (2024) analyzed reporting guidelines in medical AI and highlighted the importance of concordance to ensure the reliability and safety of AI applications in healthcare. The study emphasized the need for common standards and rigorous evaluation to maintain the quality of AI-generated medical information. Our analysis found that the superior concordance of Gemini 1.5 Pro was evident in diagnosis-related decisions, including the role of surgical lung biopsy and genomic classifier testing. In treatment-related responses, Gemini 1.5 Pro also provided more guideline-consistent recommendations, particularly in areas such as antacid therapy and antireflux surgery. These findings highlight the variability in AI-generated medical content and reinforce the need for human oversight when integrating AI tools in clinical workflows.

### 4.1 Limitations and future directions

While this study provides a structured and systematic evaluation of AI-generated medical information, certain limitations must be acknowledged. First, the analysis was limited to a predefined set of 23 questions, which, while comprehensive, may not fully capture the breadth of inquiries encountered in real-world clinical practice. Future research should expand the question pool to assess LLM performance in broader and more nuanced clinical scenarios. Second, the evaluation relied on expert assessments, which, despite efforts to standardize the rating process, remain inherently subjective. Finally, the static nature of AI model evaluation presents another limitation. As LLMs undergo continuous updates and refinements, their performance may improve over time.

Future studies should adopt a longitudinal approach to track improvements in AI-generated medical content and assess how well these models adapt to new clinical guidelines and emerging research (Bajwa et al., 2021). Another area for further investigation is the integration of AI-generated content into clinical workflows (Dossabhoy et al., 2023). While LLMs hold promise in enhancing medical decision-making and patient education, the potential risks associated with misinformation, bias, and lack of transparency must be addressed (Zhui et al., 2024). Evaluating how AI-generated responses are interpreted and utilized by healthcare providers and patients in real-world settings will be vital in determining their ultimate utility.

## 5 Conclusion

This study provides a comparative assessment of ChatGPT-4 and Gemini 1.5 Pro in generating medical information on IPF, with a particular focus on concordance with clinical guidelines. While both models demonstrated the ability to generate partially sufficient information, Gemini 1.5 Pro exhibited significantly higher concordance with established guidelines compared to ChatGPT-4. The findings highlight the ongoing need for improving AI-generated medical content, with a focus on enhancing accuracy, citation transparency, and accessibility. Given the potential role of LLMs in clinical decision support and patient education, further research should explore methods to optimize AI models and their integration into medical practice ensuring that AI-generated responses are aligned with real-time, evidence-based clinical guidelines, and are useful and comprehensible for patients.

## Data Availability

The raw data supporting the conclusions of this article will be made available by the authors, without undue reservation.

## Footnotes

^1^ https://goodcalculators.com/flesch-kincaid-calculator/
