# Supplementary material for: Evaluation of large language model-generated medical information on idiopathic pulmonary fibrosis
Source: Front Artif Intell. 2025 Sep 24;8:1618378. doi: 10.3389/frai.2025.1618378 (PMC12504196; doi:10.3389/frai.2025.1618378)
Supplement: Supplementary file 1 [file Table_1.docx]

**Supplementary Table S1. Initial and Final Set of Questions Derived from Idiopathic Pulmonary Fibrosis Clinical Practice Guidelines for Evaluation by Large Language Models**

|  | **Initial questions extracted from the Official ATS/ERS/JRS/ALAT Clinical Practice Guideline** | **Final questions** |
| --- | --- | --- |
| 1 | Should Patients with Newly Detected Interstitial Lung Disease (ILD) of Unknown Cause Who Are Clinically Suspected of Having Idiopathic Pulmonary Fibrosis (IPF) Undergo a Detailed, Prompted History of Medication Use and Environmental Exposures at Home, Work, and Other Places the Patient Frequently Visits to Exclude Potential Causes of the Interstitial Lung Disease (ILD)? | What is “clinically suspected idiopathic pulmonary fibrosis”? |
| 2 | Should Patients with Newly Detected Interstitial Lung Disease (ILD) of Unknown Cause Who Are Clinically Suspected of Having Idiopathic Pulmonary Fibrosis (IPF) Undergo Serological Testing to Exclude Connective Tissue Diseases as Potential Causes of the Interstitial Lung Disease (ILD)? | What does “likely idiopathic pulmonary fibrosis” mean? |
| 3 | Should Patients with Newly Detected Interstitial Lung Disease (ILD) of Unknown Cause Who Are Clinically Suspected of Having Idiopathic Pulmonary Fibrosis (IPF) Undergo Cellular Analysis of Their Bronchoalveolar Lavage (BAL) Fluid? | What are the criteria for indeterminate idiopathic pulmonary fibrosis? |
| 4 | For Patients with Newly Detected Interstitial Lung Disease (ILD) of Unknown Cause Who Are Clinically Suspected of Having Idiopathic Pulmonary Fibrosis (IPF), Should Surgical Lung Biopsy (SBL) Be Performed to Ascertain the Histopathology Diagnosis of Usual Interstitial Pneumonia (UIP) Pattern? | Should patients with newly detected interstitial lung disease of unknown cause who are clinically suspected of having idiopathic pulmonary fibrosis undergo serological testing to exclude connective tissue diseases as a potential cause of their interstitial lung disease? |
| 5 | For Patients with Newly Detected Interstitial Lung Disease (ILD) of Unknown Cause Who Are Clinically Suspected of Having Idiopathic Pulmonary Fibrosis (IPF), Is Transbronchial Lung Biopsy (TBBx) a Reasonable Alternative to Surgical Lung Biopsy (SBL) to Ascertain the Histopathology Diagnosis of Usual Interstitial Pneumonia (UIP) Pattern? | What autoimmune serologies should be performed in a patient with suspected IPF? |
| 6 | Should Patients with Newly Detected Interstitial Lung Disease (ILD) of Unknown Cause Who Are Clinically Suspected of Having Idiopathic Pulmonary Fibrosis (IPF), Be the Subject of Multidisciplinary Discussion for Decision-making? | Should patients with newly detected interstitial lung disease of unknown cause who are clinically suspected of having idiopathic pulmonary fibrosis be the subject of multidisciplinary discussion for decision-making? |
| 7 | Should Patients with Newly Detected Interstitial Lung Disease (ILD) of Unknown Cause Who Are Clinically Suspected of Having Idiopathic Pulmonary Fibrosis (IPF) Undergo Serum Biomarker (MMP-7, SPD, CCL-18, KL-6) Measurement for the Purpose of Diagnosis? | Can multidisciplinary discussion be used in some clinical settings to make a diagnosis of idiopathic pulmonary fibrosis in patients with a radiological pattern of probable usual interstitial pneumonia without confirmation by lung biopsy? |
| 8 | For Patients With Newly Detected Interstitial Lung Disease (ILD) Of Apparently Unknown Cause Who Are Clinically Suspected Of Having Idiopathic Pulmonary Fibrosis (IPF) And Have A High-Resolution Computed Tomography (HRCT) Pattern Of Probable Usual Interstitial Pneumonia (UIP), Indeterminate For Usual Interstitial Pneumonia (UIP), Or An Alternative Diagnosis, Is There Any Suggestion Of Cellular Analysis Of Their Bronchoalveolar Lavage (BAL)? | Should patients with newly detected interstitial lung disease of unknown cause who are clinically suspected of having idiopathic pulmonary fibrosis undergo cellular analysis of their bronchoalveolar fluid? |
| 9 | For Patients With Newly Detected Interstitial Lung Disease (ILD) Of Apparently Unknown Cause Who Are Clinically Suspected Of Having Idiopathic Pulmonary Fibrosis (IPF) And Have A High-Resolution Computed Tomography (HRCT) Pattern Of Probable Usual Interstitial Pneumonia (UIP), Indeterminate For Usual Interstitial Pneumonia (UIP), Or An Alternative Diagnosis, Is There A Suggestion For Surgical Lung Biopsy (SLB)? | For patients with newly detected interstitial lung disease of unknown cause who are clinically suspected of having idiopathic pulmonary fibrosis, should surgical lung biopsy be performed to ascertain the histopathology pattern of usual interstitial pneumonia? |
| 10 | For Patients With Newly Detected Interstitial Lung Disease (ILD) Of Apparently Unknown Cause Who Are Clinically Suspected Of Having Idiopathic Pulmonary Fibrosis (IPF) And Have A High-Resolution Computed Tomography (HRCT) Pattern Of Probable Usual Interstitial Pneumonia (UIP), Indeterminate For Usual Interstitial Pneumonia (UIP), Or An Alternative Diagnosis, Is There Any Recommendation For Transbronchial Lung Cryobiopsy (TBLC)? | For patients with newly detected interstitial lung disease of unknown cause who are clinically suspected of having idiopathic pulmonary fibrosis, is transbronchial lung cryobiopsy a reasonable alternative to surgical lung biopsy to ascertain the histopathology pattern of usual interstitial pneumonia? |
| 11 | For Patients With Newly Detected Interstitial Lung Disease (ILD) Of Apparently Unknown Cause Who Are Clinically Suspected Of Having Idiopathic Pulmonary Fibrosis (IPF) And Have A High-Resolution Computed Tomography (HRCT) Pattern Of Usual Interstitial Pneumonia (UIP), Is There Any Recommendation To Perform Surgical Lung Biopsy (SLB)? | Should patients who are clinically suspected of having idiopathic pulmonary fibrosis and have nondiagnostic findings in a transbronchial lung cryobiopsy undergo surgical lung biopsy? |
| 12 | For Patients With Newly Detected Interstitial Lung Disease (ILD) Of Apparently Unknown Cause Who Are Clinically Suspected Of Having Idiopathic Pulmonary Fibrosis (IPF) And Have An High-Resolution Computed Tomography (HRCT) Pattern Of Usual Interstitial Pneumonia (UIP), Is There Any Recommendation To Perform Transbronchial Lung Cryobiopsy (TBLC)? | Should genomic classifier testing be performed for the purpose of identifying usual interstitial pneumonia in patients with interstitial lung disease of undetermined type who are undergoing any form of lung biopsy? |
| 13 | For Patients With Newly Detected Interstitial Lung Disease (ILD) Of Apparently Unknown Cause Who Are Clinically Suspected Of Having Idiopathic Pulmonary Fibrosis (IPF), Is There Any Recommendation About Measuring Serum MMP (Matrix Metalloproteinase)-7, SPD (Surfactant Protein D), CCL (Chemokine Ligand)-18, Or KL (Krebs Von Den Lungen)-6 For The Purpose Of Distinguishing IPF From Other ILDs? | Should patients with idiopathic pulmonary fibrosis and mild to moderate impairment in pulmonary function be treated with pirfenidone as a first line treatment? |
| 14 | Should Patients With Idiopathic Pulmonary Fibrosis (IPF) And Mild To Moderate Impairment In Pulmonary Function Be Treated With Pirfenidone As a First Line Treatment? | Should patients with idiopathic pulmonary fibrosis and mild to moderate impairment in pulmonary function be treated with nintedanib as a first line treatment? |
| 15 | Should Patients With Idiopathic Pulmonary Fibrosis (IPF) And Mild Impairment In Lung Function Or Advanced Disease Benefit from the Use of Antifibrotic Medications Such As Pirfenidone Or Nintedanib? | When should patients with idiopathic pulmonary fibrosis be treated with supplemental O2? |
| 16 | Should Patients With Idiopathic Pulmonary Fibrosis (IPF) and Acute Exacerbation of their Disease Be Treated With Corticosteroids? | Should patients with idiopathic pulmonary fibrosis and confirmed gastroesophageal reflux, with or without symptoms of gastroesophageal reflux disease be treated with antacid medications to improve respiratory outcomes? |
| 17 | Should Patients With Idiopathic Pulmonary Fibrosis (IPF) And Chronic Cough Be Considered For Treatment With Corticosteroids And Thalidomide? | Should patients with idiopathic pulmonary fibrosis and confirmed gastroesophageal reflux, with or without symptoms of gastroesophageal reflux disease, be referred for antireflux surgery to improve respiratory outcomes? |
| 18 | Should Patients With Idiopathic Pulmonary Fibrosis (IPF) And Resting Hypoxemia Be Treated With Supplemental O2? | Which patients with idiopathic pulmonary fibrosis should be referred for pulmonary rehabilitation? |
| 19 | Should Patients With Idiopathic Pulmonary Fibrosis (IPF) And Gastroesophageal Reflux (GER), With Or Without Symptoms of Gastroesophageal Reflux Disease (GERD), Be Referred For Antireflux Surgery For The Purpose Of Improving Respiratory Outcomes? | How frequently should patients with idiopathic pulmonary fibrosis undergo high-resolution computed tomography of the chest for monitoring purposes? |
| 20 | Should Patients With Idiopathic Pulmonary Fibrosis and Confirmed Gastroesophageal Reflux (GER), With Or Without Symptoms of Gastroesophageal Reflux Disease (GERD), Be Treated With Antacid Medication To Improve Respiratory Outcomes? | How frequently should patients with idiopathic pulmonary fibrosis undergo pulmonary function testing with spirometry and DLCO? |
| 21 | Should Patients With Idiopathic Pulmonary Fibrosis (IPF) At Increased Risk Of Mortality Should Be Referred For Lung Transplantation At Diagnosis? | Which patients with idiopathic pulmonary fibrosis should be referred for lung transplantation? |
| 22 | Should Patients With Idiopathic Pulmonary Fibrosis (IPF) Be Considered For An Annual High-Resolution Computed Tomography (HRCT) If There Is Clinical Suspicion Of Worsening Or Risk Of Lung Cancer? | Should patients with acute exacerbation of idiopathic pulmonary fibrosis be treated with corticosteroids? |
| 23 | Should Patients With Idiopathic Pulmonary Fibrosis (IPF) Be Considered For A CT Pulmonary Angiogram If There Is A Clinical Concern For Pulmonary Embolism? | Should patients with idiopathic pulmonary fibrosis and respiratory failure be treated with mechanical ventilation? |
| 24 | Should Patients With Idiopathic Pulmonary Fibrosis (IPF) be Considered To Do A Pulmonary Function Testing And The 6-Minute-Walk Test Every 4–6 Months Or Sooner If Clinically Indicated? |  |
