# Supplementary material for: Evaluation of large language model-generated medical information on idiopathic pulmonary fibrosis
Source: Front Artif Intell. 2025 Sep 24;8:1618378. doi: 10.3389/frai.2025.1618378 (PMC12504196; doi:10.3389/frai.2025.1618378)
Supplement: Supplementary file 2 [file Table_2.docx]

**Supplementary Table S2. Consistency with guidelines and Content Analysis of ChatGPT-4 and Gemini 1.5 Pro responses to questions about Idiopathic Pulmonary Fibrosis.**

| Category | Question | Guideline recommendation | ChatGPT-4 Answer | ChatGPT-4 Concordance with Guidelines  Score, Median (IQR) | Gemini 1.5 Pro Answer | Gemini 1-5 Pro Concordance with Guidelines  Score, Median (IQR) |
| --- | --- | --- | --- | --- | --- | --- |
| Definition | 1. What is “clinically suspected idiopathic pulmonary fibrosis”? | “Clinically suspected of having IPF” is defined as unexplained patterns of bilateral pulmonary fibrosis on chest radiography or chest computed tomography, bibasilar inspiratory crackles, and age >60 years | Clinically suspected idiopathic pulmonary fibrosis (IPF) refers to a situation where a physician suspects a patient has IPF based on clinical evaluation, which includes symptoms, physical examination findings, and often imaging studies such as high-resolution computed tomography (HRCT) scans of the chest. IPF is a type of chronic, progressive lung disease characterized by fibrosis (scarring) of the lung tissue, leading to a gradual decline in lung function. The term "idiopathic" means that the cause of the disease is unknown.  The diagnosis of IPF often requires the exclusion of other potential causes of interstitial lung disease (ILD), such as environmental exposures, medications, and other systemic diseases. Confirming IPF typically involves a combination of clinical assessment, imaging, pulmonary function tests, and sometimes a lung biopsy. | 2.5 (2.25), Partially consistent | Clinically suspected idiopathic pulmonary fibrosis (IPF) refers to the initial suspicion of IPF in a patient based on their clinical presentation, typically including symptoms like shortness of breath and a dry cough, and characteristic findings on imaging tests like high-resolution CT (HRCT). Symptoms: Progressive shortness of breath (dyspnea), especially with exertion, and a persistent dry cough. Physical examination: Fine crackling sounds (Velcro crackles) heard through a stethoscope at the base of the lungs. Imaging: HRCT findings showing a pattern of fibrosis in the lungs, typically in the lower lobes and outer edges (peripheral), with honeycombing (small cystic spaces) and traction bronchiectasis (widened airways). It is important to note that clinical suspicion alone is not enough to diagnose IPF definitively. A multidisciplinary team approach, including pulmonologists, radiologists, and pathologists, is usually needed to confirm the diagnosis. This often involves further investigations like lung biopsies. | 3 (1), Mostly consistent |
|  | 2. What does “likely idiopathic pulmonary fibrosis” mean? | IPF is the likely diagnosis when any of the following features are present: 1) moderate to severe traction bronchiectasis and/or bronchiolectasis (defined as mild traction bronchiectasis and/or bronchiolectasis in four or more lobes, including the lingula as a lobe, or moderate to severe traction bronchiectasis in two or more lobes) in a man >50 years old or in a woman >60 yr old, 2) extensive (>30%) reticulation on HRCT and age>70 yr, 3) increased neutrophils and/or absence of lymphocytosis in BAL fluid, and 4) multidisciplinary discussion produces a confident diagnosis of IPF. | "Likely idiopathic pulmonary fibrosis" (IPF) refers to a probable diagnosis of a specific type of lung disease characterized by progressive scarring (fibrosis) of the lung tissue without a known cause (idiopathic). The term "likely" indicates that, based on the available medical evidence and clinical evaluation, IPF is the probable diagnosis, though it may not be definitively confirmed.  IPF causes thickening and stiffening of the lung tissue, which leads to a decline in lung function over time. Common symptoms include shortness of breath, a persistent dry cough, and fatigue. The exact cause of IPF is unknown, which is why it is termed "idiopathic."  For a detailed reference, you can refer to resources such as the Mayo Clinic or the American Lung Association: | 2.5 (2.25), Partially consistent | "Likely idiopathic pulmonary fibrosis" means that a doctor suspects a patient has pulmonary fibrosis (PF), but the exact cause is unknown. Idiopathic means "of unknown cause." Pulmonary fibrosis is a serious lung disease that occurs when lung tissue becomes damaged and scarred. This thickened, stiff tissue makes it more difficult for your lungs to work properly. As pulmonary fibrosis worsens, you become progressively more short of breath.  There are many different types of pulmonary fibrosis, each with different causes. However, in many cases, the cause is never found. This is called idiopathic pulmonary fibrosis (IPF).  The diagnosis of "likely IPF" is usually made based on a combination of factors, including:  Symptoms: The most common symptoms of IPF are shortness of breath and a dry cough. Medical history: A history of exposure to certain environmental or occupational hazards, such as asbestos or silica dust, can increase the risk of developing PF. Imaging tests: A high-resolution CT scan (HRCT) of the chest can show characteristic patterns of scarring in the lungs that are suggestive of IPF. Lung function tests: These tests measure how well your lungs are working. Lung biopsy: In some cases, a small sample of lung tissue may be removed for examination under a microscope. This can help to confirm the diagnosis of IPF. If you have been diagnosed with "likely IPF," it is important to talk to your doctor about the best treatment options for you. There is no cure for IPF, but there are treatments that can help to slow the progression of the disease and improve your quality of life. | 2.5 (3), Partially consistent |
| Diagnosis | 3. What are the criteria for indeterminate idiopathic pulmonary fibrosis? | Indeterminate for IPF is considered when 1) without an adequate biopsy remains indeterminate and 2) with an adequate biopsy may be reclassified to a more specific diagnosis after multidisciplinary discussion and/or additional consultation. | Indeterminate idiopathic pulmonary fibrosis (IPF) refers to cases where the diagnosis is unclear despite extensive evaluation. The criteria for this category are outlined in guidelines such as those from the American Thoracic Society (ATS), European Respiratory Society (ERS), Japanese Respiratory Society (JRS), and Latin American Thoracic Association (ALAT).  The 2018 ATS/ERS/JRS/ALAT guidelines provide a framework for diagnosing IPF and recognizing indeterminate cases. According to these guidelines, the criteria for indeterminate IPF include:  Clinical Presentation:  Presence of symptoms such as chronic cough and dyspnea. Age typically over 50 years. Radiological Findings:  High-resolution CT (HRCT) showing a pattern that is not definitive for usual interstitial pneumonia (UIP) but also not characteristic of other known causes of interstitial lung disease (ILD). HRCT may show some features suggestive of UIP but lacking the typical honeycombing. Histopathological Findings (if lung biopsy is performed):  Lung biopsy showing fibrosis with features that are not sufficient to meet the criteria for UIP. Presence of fibroblastic foci, architectural distortion, and patchy involvement of lung parenchyma. Exclusion of Other Causes:  Comprehensive evaluation to exclude other known causes of ILD, such as connective tissue diseases, drug-induced lung disease, and environmental exposures. Multidisciplinary Discussion:  A multidisciplinary team (MDT) including pulmonologists, radiologists, and pathologists should review the case to consider all available data and decide if the diagnosis of IPF is indeterminate. For a detailed and authoritative reference, you can consult the 2018 ATS/ERS/JRS/ALAT Clinical Practice Guideline on the diagnosis of idiopathic pulmonary fibrosis. | 3 (0.5), Mostly consistent | Idiopathic Pulmonary Fibrosis (IPF) is a chronic, progressive lung disease of unknown cause. It is characterized by scarring (fibrosis) of the lungs, leading to difficulty breathing.  The diagnosis of IPF relies on a multidisciplinary approach, including clinical, radiological, and sometimes histopathological (tissue biopsy) findings.  The 2018 international guidelines for diagnosing IPF classify high-resolution computed tomography (HRCT) patterns into four categories:  1. Usual Interstitial Pneumonia (UIP): This pattern is highly specific for IPF and is characterized by specific features like subpleural and basal predominance, reticular opacities, honeycombing, and traction bronchiectasis. 2. Probable UIP: This pattern has most of the features of UIP but may lack some of the key characteristics or have mild inconsistencies. 3. Indeterminate for UIP: This pattern has features of fibrosis but does not meet the criteria for UIP or have features suggestive of an alternative diagnosis. It may include findings like: Mild or patchy fibrosis Lack of honeycombing Presence of ground-glass opacities Findings not typical of other ILDs 4. Alternative Diagnosis: This pattern suggests an alternative diagnosis other than IPF, such as hypersensitivity pneumonitis or nonspecific interstitial pneumonia. Indeterminate for UIP is the pattern you are asking about. It is important to note that this is not a definitive diagnosis of IPF, and further investigation is usually needed. This may involve additional clinical evaluation, lung function tests, serological tests for connective tissue diseases, and sometimes a surgical lung biopsy. | 2.5 (1.5), Partially consistent |
|  | 4. Should patients with newly detected interstitial lung disease of unknown cause who are clinically suspected of having idiopathic pulmonary fibrosis undergo serological testing to exclude connective tissue diseases as a potential cause of their interstitial lung disease? | Diagnosis of IPF mandates exclusion of other causes of ILD, including CTD-ILD. The guideline panel concluded that foregoing serological testing was not a reasonable alternative. Therefore, a motherhood statement was made to perform routine serological testing in all patients with newly identified ILD. | Yes, patients with newly detected interstitial lung disease (ILD) of unknown cause who are clinically suspected of having idiopathic pulmonary fibrosis (IPF) should undergo serological testing to exclude connective tissue diseases (CTDs) as a potential cause. This recommendation is based on the understanding that some connective tissue diseases can present with ILD as a prominent or sole manifestation, and differentiating these from IPF is crucial for guiding appropriate management and treatment. | 3.5 (1.25), Mostly consistent | Yes, patients with newly detected interstitial lung disease of unknown cause who are clinically suspected of having idiopathic pulmonary fibrosis should undergo serological testing to exclude connective tissue diseases (CTD) as a potential cause of their interstitial lung disease.  Rationale:  Overlapping clinical and radiological features: CTD-associated interstitial lung disease (CTD-ILD) can present with similar clinical and radiological manifestations as idiopathic pulmonary fibrosis (IPF). Differentiating between these conditions is crucial, as treatment approaches and prognoses can vary significantly. Impact on management: Early identification of a CTD-ILD can lead to targeted immunosuppressive therapy, which may improve lung function and clinical outcomes. Misdiagnosing a CTD-ILD as IPF could result in delayed or inappropriate treatment. Prevalence of CTD-ILD: A significant proportion of patients initially diagnosed with IPF are later found to have CTD-ILD, highlighting the importance of thorough evaluation for CTD. | 3 (1), Mostly consistent |
|  | 5. What autoimmune serologies should be performed in a patient with suspected IPF? | The majority of panelists acknowledged routinely testing for CRP (C-reactive protein), erythrocyte sedimentation rate, antinuclear antibodies (by immunofluorescence), rheumatoid factor, myositis panel, and anti–cyclic citrullinated peptide. Other detailed tests are performed on a case-by-case basis according to associated symptoms and signs. | For a patient with suspected Idiopathic Pulmonary Fibrosis (IPF), it's important to evaluate for autoimmune diseases that can present with similar pulmonary manifestations. Commonly performed autoimmune serologies include:  Antinuclear Antibody (ANA) Rheumatoid Factor (RF) Anti-Cyclic Citrullinated Peptide (Anti-CCP) Antibodies Anti-Double Stranded DNA (Anti-dsDNA) Extractable Nuclear Antigens (ENAs) Anti-Smith (Anti-Sm) Anti-Ribonucleoprotein (Anti-RNP) Anti-Sjögren's-Syndrome-Related Antigen A (Anti-SSA/Ro) Anti-Sjögren's-Syndrome-Related Antigen B (Anti-SSB/La) Anti-Scleroderma-70 (Anti-Scl-70) Myositis-Specific Antibodies Anti-Jo-1 Anti-Mi-2 These tests help to rule out connective tissue diseases, such as systemic lupus erythematosus, rheumatoid arthritis, systemic sclerosis, and others, which can mimic or coexist with IPF.  For reference, you can check the guidelines published by the American Thoracic Society and the European Respiratory Society in their joint statement on the diagnosis and management of idiopathic pulmonary fibrosis: | 3 (1.25), Mostly consistent | In a patient with suspected idiopathic pulmonary fibrosis (IPF), a thorough evaluation for connective tissue disease (CTD) is essential, as CTD-related interstitial lung disease can mimic IPF. The following autoimmune serologies are recommended: Antinuclear antibodies (ANA): A positive ANA test is a hallmark of many autoimmune diseases. However, a positive ANA alone is not sufficient for a CTD diagnosis. Rheumatoid factor (RF): This antibody is associated with rheumatoid arthritis but can also be present in other CTDs. Anti-cyclic citrullinated peptide antibodies (anti-CCP): These antibodies are more specific for rheumatoid arthritis than RF. Antineutrophil cytoplasmic antibodies (ANCA): These antibodies are associated with vasculitis, a group of diseases that can affect the lungs. Anti-Jo-1 antibodies: These antibodies are specific for a type of inflammatory myopathy called polymyositis, which can also affect the lungs. Anti-Scl-70 antibodies: These antibodies are specific for systemic sclerosis (scleroderma), which can cause lung fibrosis. Anti-SSA/Ro and anti-SSB/La antibodies: These antibodies are associated with Sjögren's syndrome, which can also affect the lungs. It is important to note that a negative serology does not exclude CTD, and a positive serology does not confirm it. The results of serologic testing should always be interpreted in the context of the patient's clinical presentation and other diagnostic findings. | 3 (1), Mostly consistent |
|  | 6. Should patients with newly detected interstitial lung disease of unknown cause who are clinically suspected of having idiopathic pulmonary fibrosis be the subject of multidisciplinary discussion for decision-making? | The guideline panel agreed that MDD is preferred, because the notion that as many as 300 patients may be subject to incorrect therapy, delayed therapy, or unnecessary additional diagnostic testing was deemed unacceptable. The panel believes the benefit of MDD is greatest when the HRCT pattern is probable UIP, indeterminate for UIP, or an alternative diagnosis, or when there exist discordant clinical, radiologic, and/or histologic data. For patients with newly detected ILD of apparently unknown cause who are clinically suspected of having IPF, we suggest MDD for diagnostic decision making | Yes, patients with newly detected interstitial lung disease (ILD) of unknown cause who are clinically suspected of having idiopathic pulmonary fibrosis (IPF) should indeed be the subject of multidisciplinary discussion for decision-making. Multidisciplinary discussions typically involve pulmonologists, radiologists, pathologists, and other healthcare professionals to ensure a comprehensive evaluation and accurate diagnosis. | 3.5 (1), Mostly consistent | Yes, patients with newly detected interstitial lung disease of unknown cause who are clinically suspected of having idiopathic pulmonary fibrosis should be the subject of multidisciplinary discussion (MDD) for decision-making. | 3.5 (1), Mostly consistent |
|  | 7. Can multidisciplinary discussion be used in some clinical settings to make a diagnosis of idiopathic pulmonary fibrosis in patients with a radiological pattern of probable usual interstitial pneumonia without confirmation by lung biopsy? | Patients with a radiological pattern of probable usual interstitial pneumonia (UIP) can receive a diagnosis of IPF after multidisciplinary discussion (MDD) without confirmation by lung biopsy in the appropriate clinical setting (e.g., 60 yr old, male, smoker). | Yes, multidisciplinary discussion (MDD) can be used in clinical settings to diagnose idiopathic pulmonary fibrosis (IPF) in patients who present with a radiological pattern of probable usual interstitial pneumonia (UIP) without requiring a lung biopsy. This approach leverages the expertise of various specialists, including radiologists, pulmonologists, and pathologists, to interpret clinical, radiologic, and, when available, pathological data comprehensively.  A reference supporting this practice can be found in the official guidelines:  Raghu G, Remy-Jardin M, Myers JL, et al. Diagnosis of idiopathic pulmonary fibrosis. An official ATS/ERS/JRS/ALAT clinical practice guideline. Am J Respir Crit Care Med. 2018 Sep 1;198(5). doi: 10.1164/rccm.201807-1255ST.  This guideline highlights the importance of a multidisciplinary approach in the diagnosis of IPF, especially in cases where the radiological findings are suggestive but not definitive, and a lung biopsy is not feasible or recommended. | 3 (1.25), Mostly consistent | Yes, in some clinical settings, multidisciplinary discussion (MDD) can be used to diagnose idiopathic pulmonary fibrosis (IPF) in patients with a radiological pattern of probable usual interstitial pneumonia (UIP) on high-resolution computed tomography (HRCT) without a lung biopsy. Reference: Diagnosis of Idiopathic Pulmonary Fibrosis. An Official ATS/ERS/JRS/ALAT Clinical Practice Guideline: This guideline states that in cases where HRCT shows a pattern consistent with probable UIP, and clinical context supports IPF, a multidisciplinary team of pulmonologists, radiologists, and pathologists can discuss the case and reach a diagnosis without the need for lung biopsy. This approach is particularly useful when the risks of biopsy outweigh the benefits or when the patient is not a surgical candidate. Important Considerations:  Expertise: The MDD team should have extensive experience in diagnosing and managing ILDs. Clinical Context: The patient's medical history, symptoms, and other relevant factors should be careMostly considered. Uncertainty: If there is significant uncertainty after the MDD, a lung biopsy may still be recommended for a definitive diagnosis. Please note: This information is for educational purposes only and should not be taken as medical advice. Always consult witha healthcare professional for any health concerns. | 3 (1), Mostly consistent |
|  | 8. Should patients with newly detected interstitial lung disease of unknown cause who are clinically suspected of having idiopathic pulmonary fibrosis undergo cellular analysis of their bronchoalveolar fluid? | For patients with newly detected ILD of apparently unknown cause who are clinically suspected of having IPF and have an HRCT pattern of probable UIP, indeterminate for UIP, or an alternative diagnosis, we suggest cellular analysis of their BAL fluid. There were some strong dissenting opinions, but there was general agreement that BAL is appropriate when the radiologic differential diagnosis includes eosinophilic pneumonia, sarcoidosis, or infection. In contrast, the panel concluded that alternative diagnoses that can be excluded by BAL cellular analysis are sufficiently rare in patients who have an HRCT pattern of UIP that the downsides of the procedure typically outweigh the upsides in these patients. | Yes, patients with newly detected interstitial lung disease (ILD) of unknown cause who are clinically suspected of having idiopathic pulmonary fibrosis (IPF) should undergo cellular analysis of their bronchoalveolar lavage (BAL) fluid. This is recommended to help differentiate IPF from other types of ILD, as the cellular profile in BAL fluid can provide valuable diagnostic information. For instance, an increased number of neutrophils and eosinophils in BAL fluid can suggest other diagnoses like hypersensitivity pneumonitis, eosinophilic pneumonia, or organizing pneumonia. | 2.5 (2.25), Partially consistent | Yes, patients with newly detected interstitial lung disease of unknown cause and a clinical suspicion of idiopathic pulmonary fibrosis (IPF) should undergo bronchoalveolar lavage (BAL) with cellular analysis.  Rationale:  Differential Diagnosis: BAL can help differentiate IPF from other interstitial lung diseases (ILDs) that may have similar clinical and radiological features. The cellular profile of the bronchoalveolar fluid can provide clues about the underlying disease process. Exclusion of Other Conditions: BAL can help rule out other conditions that can mimic IPF, such as hypersensitivity pneumonitis, sarcoidosis, or cryptogenic organizing pneumonia, which may have different treatment approaches. Prognostic Information: In some cases, the cellular profile of the BAL fluid may provide prognostic information regarding the progression of IPF. | 3 (1.25), Mostly consistent |
|  | 9. For patients with newly detected interstitial lung disease of unknown cause who are clinically suspected of having idiopathic pulmonary fibrosis, should surgical lung biopsy be performed to ascertain the histopathology pattern of usual interstitial pneumonia? | For patients with newly detected ILD of apparently unknown cause who are clinically suspected of having IPF and have an HRCT pattern of UIP, we recommend NOT performing SLB. Because the likelihood of finding an etiology other than UIP is small in such patients, SLB is best considered confirmatory and, therefore, was judged by the panel to not be worth the risk of complications. | In patients with newly detected interstitial lung disease (ILD) of unknown cause who are clinically suspected of having idiopathic pulmonary fibrosis (IPF), surgical lung biopsy is often recommended to confirm the histopathology pattern of usual interstitial pneumonia (UIP). This is because the diagnosis of IPF typically requires a multidisciplinary approach, and histopathological confirmation of UIP can provide critical information for accurate diagnosis and management.  The 2018 clinical practice guideline by the American Thoracic Society (ATS), European Respiratory Society (ERS), Japanese Respiratory Society (JRS), and Latin American Thoracic Society (ALAT) provides guidance on this topic. It suggests that in patients with newly detected ILD of unknown cause who are clinically suspected of having IPF, a surgical lung biopsy should be considered when the clinical and radiographic findings are not definitive for IPF. | 2.5 (2), Partially consistent | In patients with suspected idiopathic pulmonary fibrosis (IPF), the decision to perform a surgical lung biopsy (SLB) depends on the confidence level of the diagnosis based on clinical and radiological findings.  The 2018 ATS/ERS/JRS/ALAT guidelines recommend the following:  High-resolution computed tomography (HRCT) shows a definite usual interstitial pneumonia (UIP) pattern: SLB is NOT recommended as the diagnosis of IPF can be made with high confidence (>90%) based on clinical grounds alone.  HRCT shows probable, indeterminate, or alternative diagnosis patterns: SLB is generally recommended but should be considered on a case-by-case basis, taking into account individual patient factors and risks. Additional considerations: The decision to perform an SLB should be made in consultation with a multidisciplinary team experienced in IPF diagnosis and management. The risks and benefits of SLB should be careMostly discussed with the patient. Alternative diagnostic approaches, such as bronchoscopy with transbronchial biopsy, may be considered in some cases. | 3.5 (1), Mostly consistent |
|  | 10. For patients with newly detected interstitial lung disease of unknown cause who are clinically suspected of having idiopathic pulmonary fibrosis, is transbronchial lung cryobiopsy a reasonable alternative to surgical lung biopsy to ascertain the histopathology pattern of usual interstitial pneumonia? | We suggest that TBLC be regarded as an acceptable alternative to SLB for making a histopathological diagnosis in patients with ILD of undetermined type in medical centers with experience performing and interpreting TBLC (conditional recommendation, very low quality evidence). | Transbronchial lung cryobiopsy (TBLC) is increasingly considered a reasonable alternative to surgical lung biopsy (SLB) for diagnosing idiopathic pulmonary fibrosis (IPF) in patients with suspected interstitial lung disease (ILD). Studies have shown that TBLC can provide adequate tissue samples for histopathological evaluation with fewer complications compared to SLB. TBLC has been found to have a high diagnostic yield for usual interstitial pneumonia (UIP) patterns, which is crucial for diagnosing IPF.  A reference supporting this view is the study by Ravaglia et al., published in Lancet Respiratory Medicine in 2019. The study concluded that TBLC is a valuable diagnostic tool in the multidisciplinary diagnosis of ILD, offering a good balance between diagnostic accuracy and safety. | 3 (2), Mostly consistent | Yes, transbronchial lung cryobiopsy (TBLC) is considered a reasonable alternative to surgical lung biopsy (SLB) for diagnosing usual interstitial pneumonia (UIP) in patients with suspected idiopathic pulmonary fibrosis (IPF).  A recent randomized controlled trial, the COLD study, showed that a step-up strategy using TBLC followed by SLB if needed had similar diagnostic yield compared to immediate SLB. Additionally, TBLC was associated with significantly fewer adverse events and shorter hospital stays. It's important to note that this is a rapidly evolving field and the decision on which biopsy method to use should be individualized based on the patient's specific circumstances and discussed with their healthcare provider. | 3.5 (1.25), Mostly consistent |
|  | 11. Should patients who are clinically suspected of having idiopathic pulmonary fibrosis and have nondiagnostic findings in a transbronchial lung cryobiopsy undergo surgical lung biopsy? | The original question and systematic review involved the comparison of TBLC versus no TBLC (i.e., TBLC followed by SLB, if needed, vs. going directly to SLB). However, the committee concluded that the comparison had become outdated because observations published during guideline development suggest that patients who have nondiagnostic findings on TBLC are likely to also have nondiagnostic findings on SLB. | In cases where patients are clinically suspected of having idiopathic pulmonary fibrosis (IPF) and have nondiagnostic findings in a transbronchial lung cryobiopsy (TBLC), undergoing a surgical lung biopsy (SLB) is generally recommended. This recommendation is based on the need for a definitive diagnosis to guide appropriate treatment and management strategies for IPF, which can be challenging to diagnose due to overlapping features with other interstitial lung diseases. | 3 (2), Mostly consistent | Whether patients with suspected idiopathic pulmonary fibrosis (IPF) and nondiagnostic transbronchial lung cryobiopsy (TBLC) should undergo surgical lung biopsy (SLB) is a complex decision that requires a careful balance of risks and benefits.  Factors to consider:  Confidence in the clinical diagnosis: If the clinical suspicion for IPF is high based on history, physical examination, pulmonary function tests, and imaging (particularly a high-resolution CT scan showing a usual interstitial pneumonia (UIP) pattern), SLB may not be necessary. Alternative diagnoses: If there are other potential diagnoses that could be treated differently than IPF, then SLB may be necessary to rule them out. Patient's overall health and surgical risk: SLB is an invasive procedure with potential complications, so the patient's overall health and surgical risk should be assessed. Patient's preferences: The patient's values and preferences should be considered in the decision-making process. General recommendations:  High clinical suspicion for IPF and typical UIP pattern on HRCT: SLB is generally not recommended. High clinical suspicion for IPF but atypical or indeterminate HRCT pattern: SLB may be considered on a case-by-case basis. Important note: The decision to undergo SLB should be made in consultation with a pulmonologist and thoracic surgeon who can assess the individual patient's case and provide personalized recommendations. Low clinical suspicion for IPF or other potential diagnoses: SLB may be necessary. | 3.5 (1), Mostly consistent |
|  | 12. Should genomic classifier testing be performed for the purpose of identifying usual interstitial pneumonia in patients with interstitial lung disease of undetermined type who are undergoing any form of lung biopsy? | The guideline committee made no recommendation for or against genomic classifier testing, because of insufficient agreement among the committee members. | Genomic classifier testing, such as the Envisia Genomic Classifier, can be useful in identifying usual interstitial pneumonia (UIP) in patients with interstitial lung disease (ILD) of undetermined type. The utility of such testing is particularly relevant when traditional diagnostic methods, including high-resolution computed tomography (HRCT) and lung biopsy, are inconclusive. A reference supporting this practice is provided by the American Thoracic Society (ATS) in their 2022 clinical practice guideline for the diagnosis of idiopathic pulmonary fibrosis (IPF) | 2.5 (1.25), Partially consistent | The use of genomic classifier testing (GCT) to identify usual interstitial pneumonia (UIP) in patients with interstitial lung disease (ILD) of undetermined type is a developing area. While GCT has shown promise in predicting UIP patterns with high specificity, its sensitivity is moderate and it is not yet widely available.  Current clinical practice guidelines do not recommend routine use of GCT for all patients with ILD undergoing lung biopsy. However, it may be considered in specific scenarios, such as:  Patients with probable UIP pattern on imaging: GCT can help confirm the UIP pattern and potentially avoid the need for more invasive surgical lung biopsies. Patients without access to an ILD center: GCT can provide valuable information to pulmonologists who may not have specialized expertise in ILD diagnosis. Patients with high clinical suspicion of IPF: GCT can increase diagnostic confidence and potentially avoid the need for further invasive testing. It is important to note that GCT results should always be interpreted in the context of other clinical and radiological information. A multidisciplinary discussion (MDD) involving pulmonologists, radiologists, and pathologists is crucial for accurate ILD diagnosis. | 3 (1.25), Mostly consistent |
| Treatment | 13. Should patients with idiopathic pulmonary fibrosis and mild to moderate impairment in pulmonary function be treated with pirfenidone as a first line treatment? | Treatment considerations should include both pharmacological (nintedanib and pirfenidone) and nonpharmacological (oxygen supplementation if hypoxemic and/or pulmonary rehabilitation) therapies. | Yes, patients with idiopathic pulmonary fibrosis (IPF) and mild to moderate impairment in pulmonary function are often treated with pirfenidone as a first-line treatment. Pirfenidone has been shown to slow the progression of IPF, reduce lung function decline, and improve progression-free survival.  A key reference supporting this is the ASCEND trial. This study demonstrated that pirfenidone significantly reduced the decline in forced vital capacity (FVC) compared to placebo in patients with IPF, supporting its use as a first-line treatment. | 3.5 (2), Mostly consistent | Yes, pirfenidone is considered a first-line treatment for patients with idiopathic pulmonary fibrosis (IPF), even in those with mild to moderate impairment in pulmonary function.  Rationale:  Slows Disease Progression: Clinical trials have shown that pirfenidone can slow the decline in lung function and reduce the risk of acute exacerbations in patients with IPF, regardless of the initial degree of lung impairment. Improves Quality of Life: By preserving lung function, pirfenidone can help maintain patients' quality of life and functional capacity for a longer period. Limited Side Effects: Pirfenidone is generally well-tolerated, with most side effects being mild to moderate and manageable. Reference:  A good reference supporting this recommendation is:  Pirfenidone in idiopathic pulmonary fibrosis: https://erj.ersjournals.com/content/35/4/821 This article provides a comprehensive overview of the efficacy and safety of pirfenidone in IPF and supports its use as a first-line treatment option.  Important Considerations:  Individualized Treatment: The decision to initiate pirfenidone should be made on a case-by-case basis, considering the patient's overall health status, disease severity, and potential risks and benefits. Monitoring: Patients on pirfenidone should be closely monitored for side effects and response to treatment. Other Options: Nintedanib is another first-line treatment option for IPF, and the choice between pirfenidone and nintedanib may depend on individual patient factors and preferences. Please note that this information is not a substitute for professional medical advice. Consult with a healthcare provider for personalized treatment recommendations. | 4 (1), Fully consistent |
|  | 14. Should patients with idiopathic pulmonary fibrosis and mild to moderate impairment in pulmonary function be treated with nintedanib as a first line treatment? | Treatment considerations should include both pharmacological (nintedanib and pirfenidone) and nonpharmacological (oxygen supplementation if hypoxemic and/or pulmonary rehabilitation) therapies. | Yes, patients with idiopathic pulmonary fibrosis (IPF) and mild to moderate impairment in pulmonary function can be treated with nintedanib as a first-line treatment. Clinical guidelines and studies have shown that nintedanib is effective in slowing the progression of IPF.  A relevant reference for this recommendation is:  Richeldi, L., et al. (2014). "Efficacy and Safety of Nintedanib in Idiopathic Pulmonary Fibrosis." New England Journal of Medicine, 370(22), 2071-2082. Link to article This study demonstrated that nintedanib significantly reduced the annual rate of decline in forced vital capacity (FVC), a key measure of lung function, compared to placebo, indicating its effectiveness in treating IPF. | 3 (1.25), Mostly consistent | Yes, patients with idiopathic pulmonary fibrosis (IPF) and mild to moderate impairment in pulmonary function should be considered for treatment with nintedanib as a first-line therapy. Nintedanib has been shown to slow the progression of IPF, even in patients with preserved lung function (forced vital capacity [FVC] >90% predicted).  The INPULSIS trials demonstrated that nintedanib significantly reduced the annual rate of decline in FVC compared to placebo in patients with IPF, regardless of the degree of lung function impairment at baseline. A subsequent analysis of pooled data from the INPULSIS trials specifically addressed the efficacy of nintedanib in patients with preserved lung function (FVC >90% predicted). It showed that nintedanib slowed the decline in lung function to a similar extent in both subgroups (FVC >90% and ≤90% predicted).  Reference:  Richeldi L, du Bois RM, Raghu G, et al. Efficacy and safety of nintedanib in idiopathic pulmonary fibrosis. N Engl J Med. 2014;370(22):2071-2082. doi:10.1056/NEJMoa1402582  In addition, international guidelines (such as those from the American Thoracic Society, European Respiratory Society, and Japanese Respiratory Society) recommend nintedanib (along with pirfenidone) as a first-line treatment option for patients with IPF, regardless of the severity of lung function impairment.  However, the decision to initiate nintedanib should be made on an individual basis, taking into consideration the patient's specific clinical characteristics, comorbidities, and preferences. It is essential to discuss the potential benefits and risks of treatment with the patient. | 3.5 (1.25), Mostly consistent |
|  | 15. When should patients with idiopathic pulmonary fibrosis be treated with supplemental O2? | Treatment considerations should include both pharmacological (nintedanib and pirfenidone) and nonpharmacological (oxygen supplementation if hypoxemic and/or pulmonary rehabilitation) therapies. | Patients with idiopathic pulmonary fibrosis (IPF) should be treated with supplemental oxygen (O2) when they exhibit hypoxemia, particularly if their blood oxygen saturation (SpO2) falls below 88% at rest, during exertion, or during sleep. This guideline helps to manage symptoms such as shortness of breath and improve quality of life.  A reference for this recommendation is:  Raghu G, et al. (2011). An Official ATS/ERS/JRS/ALAT Statement: Idiopathic Pulmonary Fibrosis: Evidence-based Guidelines for Diagnosis and Management. American Journal of Respiratory and Critical Care Medicine, 183(6), 788-824. DOI: 10.1164/rccm.2009-040GL. This guideline outlines the management strategies for IPF, including the use of supplemental oxygen therapy in patients with significant hypoxemia. | 2.5 (1.25), Partially consistent | Patients with idiopathic pulmonary fibrosis (IPF) should be treated with supplemental oxygen when their oxygen levels fall below a certain threshold, typically when the partial pressure of oxygen in arterial blood (PaO2) is less than 55 mmHg or oxygen saturation (SaO2) is less than or equal to 88% on room air. This can be measured at rest, during exercise, or while sleeping.  Supplemental oxygen is also recommended during exertion for patients whose oxygen saturation drops below 88%. Additionally, supplemental oxygen may be considered for patients with pulmonary hypertension or right heart failure associated with IPF, even if they do not meet the above criteria. | 3 (1.25), Mostly consistent |
|  | 16. Should patients with idiopathic pulmonary fibrosis and confirmed gastroesophageal reflux, with or without symptoms of gastroesophageal reflux disease, be treated with antacid medications to improve respiratory outcomes? | In the absence of any definitive benefits, the committee voted to make a conditional recommendation against treating patients with IPF with antacid medication for the sole purpose of improving respiratory outcomes. | The use of antacid medications in patients with idiopathic pulmonary fibrosis (IPF) and gastroesophageal reflux (GER) has been a topic of interest in clinical research. Studies have investigated whether treating GER with antacid medications can improve respiratory outcomes in IPF patients.  One significant study that addresses this question is by Lee et al., published in the journal Chest in 2013. The study, titled "Antacid therapy and disease progression in idiopathic pulmonary fibrosis: an analysis of data from three randomized controlled trials," found that patients with IPF who were treated with antacid medications had a slower disease progression compared to those who were not treated with these medications. This study supports the notion that antacid treatment may be beneficial in managing disease progression in IPF patients with GER, regardless of the presence of symptomatic gastroesophageal reflux disease (GERD). | 2 (3), Partially consistent | The current evidence does not support the routine use of antacid medications to improve respiratory outcomes in patients with idiopathic pulmonary fibrosis (IPF) and confirmed gastroesophageal reflux (GER), even if they have symptoms of gastroesophageal reflux disease (GERD).  A systematic review and meta-analysis published in 2022 concluded that there is insufficient evidence to determine whether antacid medications improve respiratory outcomes in patients with IPF, most of whom did not have abnormal GER confirmed. The study emphasized the need for well-designed, adequately powered prospective studies with objective evaluation for GER to clarify the role of antacid medication in this population. Additional Considerations:  *Individualized Approach: Treatment decisions should be made on a case-by-case basis, considering the severity of GERD symptoms, the patient's overall health status, and potential risks and benefits of antacid therapy. *Alternative Therapies: Lifestyle modifications such as elevating the head of the bed, avoiding meals close to bedtime, and weight loss if appropriate may be considered as first-line interventions for GERD. *Ongoing Research: Research in this area is ongoing, and future studies may provide clearer guidance on the use of antacid medications in patients with IPF and GER. Please consult with a healthcare professional for personalized advice and treatment recommendations. | 3 (1), Mostly consistent |
|  | 17. Should patients with idiopathic pulmonary fibrosis and confirmed gastroesophageal reflux, with or without symptoms of gastroesophageal reflux disease, be referred for antireflux surgery to improve respiratory outcomes? | We suggest not referring patients with IPF for antireflux surgery for the purpose of improving respiratory outcomes | The consideration of antireflux surgery for patients with idiopathic pulmonary fibrosis (IPF) and confirmed gastroesophageal reflux (GER), regardless of the presence of gastroesophageal reflux disease (GERD) symptoms, is an area of active research. Studies suggest that GER can contribute to the progression of IPF, and managing GER might help improve respiratory outcomes.  A notable study on this topic is:  Title: "A Randomized Controlled Trial of Antireflux Surgery for Idiopathic Pulmonary Fibrosis" Authors: Raghu G, Pellegrini CA, Yow E, et al. Journal: American Journal of Respiratory and Critical Care Medicine Year: 2018 Volume: 198 Issue: 5 Pages: 635-642  Summary: This study investigated the effects of antireflux surgery on patients with IPF. The findings suggest that antireflux surgery may stabilize lung function and potentially slow disease progression in patients with IPF who have abnormal acid GER, even in the absence of typical GERD symptoms. | 2 (1.25), Partially consistent | The current evidence does not definitively recommend antireflux surgery for all patients with idiopathic pulmonary fibrosis (IPF) and confirmed gastroesophageal reflux (GER), with or without symptoms of gastroesophageal reflux disease (GERD), to improve respiratory outcomes.  A 2022 systematic review and meta-analysis published in the Annals of the American Thoracic Society concluded that there is insufficient evidence to recommend for or against antireflux surgery in patients with IPF and documented GER. The studies included in the review were limited by small sample sizes, heterogeneity, and potential biases.  While some studies have suggested potential benefits of antireflux surgery in improving respiratory function and slowing disease progression in IPF patients, more robust, randomized controlled trials are needed to establish a clear causal relationship and determine the long-term effects of surgery on respiratory outcomes.  Therefore, the decision to refer a patient with IPF and GER for antireflux surgery should be made on a case-by-case basis, considering individual factors such as the severity of GER, response to medical management, presence of GERD symptoms, and overall health status. | 3 (1), Mostly consistent |
|  | 18. Which patients with idiopathic pulmonary fibrosis should be referred for pulmonary rehabilitation? | Treatment considerations should include both pharmacological (nintedanib and pirfenidone) and nonpharmacological (oxygen supplementation and/or pulmonary rehabilitation) therapies. | Patients with idiopathic pulmonary fibrosis (IPF) who should be referred for pulmonary rehabilitation (PR) typically include those who:  Experience dyspnea or shortness of breath that limits their daily activities. Have a reduced exercise tolerance. Have stable respiratory conditions without recent exacerbations. Are motivated and able to participate in a structured rehabilitation program. A reference for this recommendation is:  Spruit, M. A., Singh, S. J., Garvey, C., ZuWallack, R., Nici, L., Rochester, C., ... & Wouters, E. F. (2013). An official American Thoracic Society/European Respiratory Society statement: key concepts and advances in pulmonary rehabilitation. American Journal of Respiratory and Critical Care Medicine, 188(8), e13-e64.  This document outlines the criteria and benefits of pulmonary rehabilitation for various respiratory conditions, including idiopathic pulmonary fibrosis. | 2.5 (1.25), Partially consistent | Pulmonary rehabilitation (PR) is recommended for all patients with idiopathic pulmonary fibrosis (IPF) who are experiencing dyspnea and/or exercise limitation, regardless of disease severity.  This recommendation is based on the following:  Current IPF guidelines: The 2022 IPF guidelines from the American Thoracic Society, European Respiratory Society, Japanese Respiratory Society, and Latin American Thoracic Association include a conditional recommendation for PR in patients with IPF. Clinical studies: Several clinical studies have shown that PR can improve exercise capacity, quality of life, and dyspnea in patients with IPF. Safety: PR is generally safe and well-tolerated in patients with IPF. | 3 (1), Mostly consistent |
|  | 19. Which patients with idiopathic pulmonary fibrosis should be referred for lung transplantation? | Patients at increased risk of mortality should be referred for lung transplantation at diagnosis. | Patients with idiopathic pulmonary fibrosis (IPF) who should be considered for lung transplantation typically include those with the following characteristics:  Severe disease: Often measured by a forced vital capacity (FVC) of less than 80% predicted or a diffusion capacity for carbon monoxide (DLCO) of less than 40% predicted. Rapid disease progression: Indicated by a significant decline in FVC (greater than 10%) or DLCO (greater than 15%) over six months. Worsening hypoxemia: Especially during exertion. Frequent hospitalizations: Due to respiratory failure or acute exacerbations. Failure of medical therapy: When standard treatments, such as antifibrotic agents (pirfenidone or nintedanib), are not effectively slowing disease progression. Referral for lung transplantation should be considered early in the disease course due to the unpredictable nature of IPF progression. | 3 (2), Mostly consistent | Patients with idiopathic pulmonary fibrosis (IPF) should be referred for lung transplantation evaluation early in their disease course. This allows for adequate time to assess eligibility, optimize health, and undergo the necessary pre-transplant evaluations.  Specific criteria for referral can vary between transplant centers, but generally include:  Progressive disease: Documented decline in lung function despite optimal medical therapy. Worsening symptoms: Increasing shortness of breath, exercise intolerance, or cough. Decline in quality of life: Significant impact on daily activities and overall well-being. Specific lung function parameters: Forced vital capacity (FVC) < 80% predicted Diffusing capacity of the lung for carbon monoxide (DLCO) < 40% predicted Additional factors that may be considered include:  Age: Generally less than 65 years old, but older patients may be considered depending on overall health and functional status. Comorbidities: The presence of other medical conditions is assessed individually to determine overall suitability for transplantation. Nutritional status: Adequate nutrition is essential for optimal surgical outcomes. Psychosocial factors: A supportive social network and good mental health are important for post-transplant recovery and adherence to complex medical regimens. Important Note: It is crucial to consult with a specialist in interstitial lung diseases and a lung transplant center to determine the most appropriate course of action for each individual patient. | 3 (0.25), Mostly consistent |
|  | 20. Should patients with acute exacerbation of idiopathic pulmonary fibrosis be treated with corticosteroids? | Acute exacerbations may be treated with corticosteroids | The use of corticosteroids in patients with acute exacerbations of idiopathic pulmonary fibrosis (IPF) is a common but controversial practice. There is no definitive evidence from randomized controlled trials supporting their efficacy, and treatment is often based on clinical judgment and experience.  A review article by Collard HR, Moore BB, Flaherty KR, et al. titled "Acute exacerbations of idiopathic pulmonary fibrosis" published in the American Journal of Respiratory and Critical Care Medicine (2007) discusses the use of corticosteroids among other treatments for acute exacerbations of IPF. The article suggests that corticosteroids are commonly used, but the evidence for their benefit is not strong, and further research is needed. | 3 (0.25), Mostly consistent | he use of corticosteroids for the treatment of acute exacerbation of idiopathic pulmonary fibrosis (AE-IPF) is a complex and controversial issue.  Current guidelines recommend the use of corticosteroids for AE-IPF, based on anecdotal reports of benefit and the high mortality rate of this condition. However, there is limited high-quality evidence to support this recommendation, and some studies suggest potential harm associated with corticosteroid use in these patients.  Potential benefits: Corticosteroids are anti-inflammatory agents that may help reduce the lung inflammation seen in AE-IPF. Some studies have reported short-term improvements in oxygenation and lung function with corticosteroid use.  Potential risks: Corticosteroids can suppress the immune system, potentially increasing the risk of infections. They can also cause other side effects such as hyperglycemia, osteoporosis, and muscle weakness. Some research suggests that corticosteroids may not improve long-term outcomes in AE-IPF and might even be associated with increased mortality.  Given the lack of definitive evidence and the potential risks, the decision to use corticosteroids in AE-IPF should be made on a case-by-case basis, considering the individual patient's characteristics, the severity of the exacerbation, and the potential benefits and risks of treatment.It's crucial to have a thorough discussion with the patient about the uncertainties surrounding corticosteroid use in AE-IPF. | 3 (0.5), Mostly consistent |
|  | 21. Should patients with idiopathic pulmonary fibrosis and respiratory failure be treated with mechanical ventilation? | Mechanical ventilation is not recommended for the majority of patients with respiratory failure. | The use of mechanical ventilation in patients with idiopathic pulmonary fibrosis (IPF) and respiratory failure is controversial. Generally, mechanical ventilation is considered a last resort due to the poor prognosis associated with IPF in such advanced stages. However, it might be used in specific circumstances, such as a reversible cause of respiratory failure or in the context of bridging to lung transplantation.  A key reference discussing this topic is:  Papiris, S. A., Triantafillidou, C., Kolilekas, L., Markoulaki, E., & Manali, E. D. (2013). "Pirfenidone and nintedanib: two new drugs for idiopathic pulmonary fibrosis." Pneumon, 26(1), 20-25.  This paper highlights the complexity of managing advanced IPF and the critical consideration of mechanical ventilation. | 3 (2), Mostly consistent | The decision to use mechanical ventilation (MV) in patients with idiopathic pulmonary fibrosis (IPF) and respiratory failure is complex and should be made on a case-by-case basis, considering individual patient factors and goals of care.  Guidelines and Studies: The 2011 international guidelines generally recommend against MV in patients with IPF and respiratory failure who are not candidates for lung transplantation. This is due to the historically poor prognosis associated with MV in this population. Several studies have reported high mortality rates (up to 87%) in IPF patients receiving MV. However, these studies were often conducted at single centers with small sample sizes and may not reflect current practices. More recent research suggests that short-term mortality in IPF patients on MV may be decreasing due to improvements in intensive care and ventilator management. However, the long-term prognosis remains poor. Factors to Consider: Patient's Wishes: The patient's preferences and goals of care should be the primary consideration. Some patients may choose to forgo MV if they understand the potential risks and benefits. Reversibility of Respiratory Failure: If the respiratory failure is due to a potentially reversible cause (e.g., infection, heart failure), MV may be considered as a temporary measure to support the patient while the underlying cause is treated. Lung Transplantation Candidacy: For patients who are candidates for lung transplantation, MV may be used as a bridge to transplant. Quality of Life: The potential impact of MV on the patient's quality of life should be careMostly considered. MV can be uncomfortable and may prolong suffering in some cases. Reference: You can find more information in this study: Mechanical ventilation in patients with idiopathic pulmonary fibrosis in Korea: a nationwide cohort study: [invalid URL removed] In conclusion: It's crucial for healthcare providers to have a comprehensive discussion with patients and their families about the risks and benefits of MV in the context of IPF and respiratory failure. This discussion should include the patient's prognosis, goals of care, and potential impact on quality of life. | 3 (1.25), Mostly consistent |
| Follow-up | 22. How frequently should patients with idiopathic pulmonary fibrosis undergo high-resolution computed tomography of the chest for monitoring purposes? | Consider annual HRCT if there is clinical suspicion of worsening or risk of lung cancer | The frequency of high-resolution computed tomography (HRCT) for monitoring patients with idiopathic pulmonary fibrosis (IPF) varies based on individual patient circumstances, disease progression, and clinical guidelines. Generally, HRCT is used at diagnosis and may be repeated every 6-12 months, or sooner if there are significant changes in symptoms or lung function. | 3 (2), Mostly consistent | There is no universally agreed-upon frequency for high-resolution computed tomography (HRCT) monitoring in patients with idiopathic pulmonary fibrosis (IPF). The optimal frequency depends on various factors, including disease severity, progression rate, treatment response, and the presence of comorbidities.  However, here are some general recommendations based on current clinical practice and expert opinions:  Baseline HRCT: All patients diagnosed with IPF should undergo a baseline HRCT to assess the extent of lung involvement and establish a reference point for future comparisons. Initial monitoring: In the first year after diagnosis, HRCT scans may be performed every 3-6 months to assess disease progression and response to treatment. Stable disease: If the disease remains stable, the frequency of HRCT scans can be reduced to every 6-12 months. Progressive disease or treatment changes: If there are signs of disease progression or changes in treatment, HRCT scans may be performed more frequently (e.g., every 3-6 months) to monitor the disease course and assess treatment response. Clinical indications: HRCT scans may also be performed at any time if there are new or worsening respiratory symptoms, or if other clinical indications arise. | 3.5 (1), Mostly consistent |
|  | 23. How frequently should patients with idiopathic pulmonary fibrosis undergo pulmonary function testing with spirometry and DLCO? | Consider pulmonary function testing and the 6-minute-walk test every 4–6 months or sooner if clinically indicated | Patients with idiopathic pulmonary fibrosis (IPF) typically undergo pulmonary function testing (PFT) with spirometry and diffusing capacity of the lungs for carbon monoxide (DLCO) every 3 to 6 months. This frequency helps monitor disease progression and response to therapy.  For a detailed reference, you can look at the guidelines from the American Thoracic Society (ATS), European Respiratory Society (ERS), Japanese Respiratory Society (JRS), and Latin American Thoracic Association (ALAT). Specifically, the 2011 ATS/ERS/JRS/ALAT guidelines on the diagnosis and management of idiopathic pulmonary fibrosis recommend regular monitoring of pulmonary function tests in patients with IPF. | 3 (2), Mostly consistent | The frequency of pulmonary function testing (PFT) with spirometry and DLCO in patients with idiopathic pulmonary fibrosis (IPF) can vary depending on several factors, including disease severity, stability, and treatment response. However, a general guideline is as follows:  Initial diagnosis: PFTs should be performed at the time of initial diagnosis to establish baseline lung function and assess the severity of the disease. Monitoring disease progression: PFTs are typically repeated every 3-6 months to monitor disease progression and assess the response to treatment. If the disease is stable, the frequency of testing may be reduced to every 6-12 months. Change in symptoms: PFTs should be performed if there is a significant change in the patient's symptoms, such as worsening shortness of breath or cough, to determine if there has been a decline in lung function. | 3.5 (1), Mostly consistent |

**Footnote**: Responses were evaluated by six independent raters across four domains (Definition, Diagnosis, Follow-up, Treatment) using a 5-point Likert-type scale (0–4), where 0 = Not consistent, 1 = Slightly consistent, 2 = Partially consistent, 3 = Mostly consistent, and 4 = Fully consistent. Median scores and interquartile ranges (IQR) were computed.
